# Origin and evolutionary plasticity of the gastric caecum in sea urchins (Echinodermata: Echinoidea)

**DOI:** 10.1186/1471-2148-10-313

**Published:** 2010-10-18

**Authors:** Alexander Ziegler, Rich Mooi, Gauthier Rolet, Chantal De Ridder

**Affiliations:** 1 Institut für Immungenetik, Charité-Universitätsmedizin Berlin, Thielallee 73, 14195 Berlin, Germany; 2 California Academy of Sciences, Golden Gate Park, 55 Music Concourse Drive, San Francisco, California, 94118, USA; 3 Laboratoire de Biologie Marine, Université Libre de Bruxelles, 50 avenue F.D. Roosevelt, 1050 Bruxelles, Belgium

## Abstract

**Background:**

The digestive tract of many metazoan invertebrates is characterized by the presence of caeca or diverticula that serve secretory and/or absorptive functions. With the development of various feeding habits, distinctive digestive organs may be present in certain taxa. This also holds true for sea urchins (Echinodermata: Echinoidea), in which a highly specialized gastric caecum can be found in members of a derived subgroup, the Irregularia (cake urchins, sea biscuits, sand dollars, heart urchins, and related forms). As such a specialized caecum has not been reported from "regular" sea urchin taxa, the aim of this study was to elucidate its evolutionary origin.

**Results:**

Using morphological data derived from dissection, magnetic resonance imaging, and extensive literature studies, we compare the digestive tract of 168 echinoid species belonging to 51 extant families. Based on a number of characters such as topography, general morphology, mesenterial suspension, and integration into the haemal system, we homologize the gastric caecum with the more or less pronounced dilation of the anterior stomach that is observed in most "regular" sea urchin taxa. In the Irregularia, a gastric caecum can be found in all taxa except in the Laganina and Scutellina. It is also undeveloped in certain spatangoid species.

**Conclusions:**

According to our findings, the sea urchin gastric caecum most likely constitutes a synapomorphy of the Euechinoidea. Its occurrence in "regular" euechinoids is linked to the presence of an additional festoon of the anterior stomach in ambulacrum III. Both structures, the additional festoon and the gastric caecum, are absent in the sister taxon to the Euechinoidea, the Cidaroida. Since the degree of specialization of the gastric caecum is most pronounced in the predominantly sediment-burrowing irregular taxa, we hypothesize that its evolution is closely linked to the development of more elaborate infaunal lifestyles. We provide a comprehensive study of the origin and evolutionary plasticity of a conspicuous digestive tract structure, the gastric caecum, in a major taxon of the extant invertebrate macrozoobenthos.

## Background

With few exceptions, metazoans possess an alimentary canal comprising a sac- or tube-like invagination of the body wall. The evolution of an internalized intestinal tract offered the possibility of digesting larger food particles [1]. The digestive system may form a simple or ramified cavity with a single aperture (as in the Cnidaria and the Platyhelminthes) or a tube with openings at its two ends that constitute a distinct mouth and anus, allowing the food to pass in one direction through a tubular system [2]. Subsequently, this has led to the specialization of entire digestive tract regions. The invertebrate gut can be subdivided into three major parts: the foregut (usually comprising mouth, pharynx, and esophagus), the midgut (crop, gizzard, and stomach), and the hindgut (intestine, rectum, and anus) [3]. In most taxa, the midgut serves as the primary site of digestion as well as nutrient absorption and is therefore often characterized by the presence of glands and caeca that serve secretory or absorptive functions.

**Figure 1:**
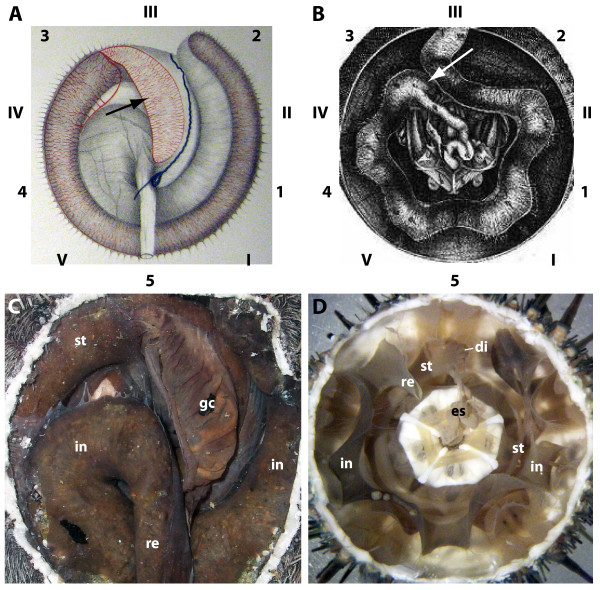
**Historic and contemporary representations of the general anatomy of the irregular and "regular" sea urchin digestive tract**. (A) and (B) constitute the first graphic representations of the gastric caecum (black arrow in A) as well as the dilation of the anterior stomach (white arrow in B). (A) *Spatangus purpureus *- aboral view, modified from Hoffmann [8]. (B) *Paracentrotus lividus *- aboral view, modified from Tiedemann [20]. Numbers indicate homologous body parts in "regular" and irregular sea urchins according to Lovén's system [29]: Roman numerals (I-V) indicate ambulacra, whereas Arabic numerals (1-5) indicate interambulacra. (C) *Spatangus purpureus *- aboral view of a dissected specimen. (D) *Paracentrotus lividus *- aboral view of a dissected specimen. di = dilation, es = esophagus, gc = gastric caecum, in = intestine, re = rectum, st = stomach. Not to scale.

In the context of the general pattern described above for typical bilaterian animals, it is important to note that even secondarily radial forms such as echinoderms tend to follow the same overall model of gut organization. Among the Echinodermata - a taxon of marine invertebrate deuterostomes - sea urchins (Echinoidea) are considered one of the best studied groups and serve as model organisms for a wide range of biological disciplines. The digestive tract of echinoids is usually subdivided into mouth, buccal cavity, pharynx, esophagus, stomach, intestine, rectum, and anus [4-6], with the mouth forming the proximal and the anus the distal segments. However, not all sea urchin taxa possess all of these gut sections and some are characterized by the presence of additional digestive tract structures such as festoons, siphons, Gregory's diverticulum, an intestinal caecum, or a gastric caecum [7]. The gastric caecum is a conspicuous organ that was first described by C.K. Hoffmann [8] in *Spatangus purpureus*, a species within the derived Spatangoida (Figure 1A), a monophyletic taxon of irregular sea urchins characterized by an infaunal lifestyle. Several spatangoid taxa have been shown to possess this large, non-contractile pouch that is connected to the anterior stomach through a slit-like opening (Figure 1C). This pouch is also well-connected to the haemal system through numerous haemal ducts within its connective tissue layer [9-12]. A number of hypotheses regarding the function of this structure in spatangoids have been presented. Some authors believed it to be a glandular organ whose secretions leak into the stomach [13], an absorptive structure [12,14], a site of microbial fermentation [15,16], or simply an organ that acts generally in digestion [17,18].

In contrast, the digestive tract in "regular" sea urchins is not characterized by the presence of such a highly specialized structure (the "regular" echinoids do not form a monophyletic group, hence the quotes; in contrast, the Irregularia is a recognized monophyletic taxon [19]). However, several authors [5,9,14,20-24] reported a more or less developed dilation at the proximal part of the anterior stomach in certain "regular" sea urchin taxa (Figure 1B, D). According to most authors, this dilation in "regular" sea urchins did not display any functional specialization and was therefore seen merely as a lateral outcrop of the stomach [5,9,22,23]. R. Koehler - who had systematically studied sea urchin internal anatomy - was presumably the first and so far the only author to briefly mention the potential homology of the dilation observed in "regular" sea urchins with the highly specialized gastric caecum found in the infaunal spatangoids and other irregular taxa in which the caecum had been described [9]. However, the precise evolutionary relationship between these structures has not yet been systematically elucidated, largely because a comprehensive analysis encompassing all major sea urchin taxa was not possible due to the lack of data. In addition, the multitude of terms assigned by several authors to the observed dilation of the sea urchin anterior stomach as well as the gastric caecum in the Irregularia has greatly complicated matters by obfuscating direct comparisons among observed occurrences (Table 1).

**Table 1:** Trilingual list of terms assigned to the pouch encountered in irregular as well as to the dilation of the anterior stomach observed in "regular" sea urchin species by various authors.

English		French	German
actinal intestinal appendage	diverticulum of the stomach	appendice	Blinddarm
anterior caecum	festoon	appendice cecal	Blindsack
blind diverticulum	first caecum	caecum	Blindsackbildung
blind gut	gastric caecum	caecum gastrique	Caecum
blindsac	intestinal appendage	caecum stomacal	Coecum
blind sac	pouch	coecum stomacal	Erstes Divertikel
caecum	sac	cul-de-sac	Divertikel
coecum	sac-like dilatation	cul-de-sac antérieur de l'intestin	Erweiterung
digestive caecum	sac-like swelling	diverticule en cul-de-sac	
dilatation	stomach caecum	diverticulum	
dilation	swelling	diverticulum intestinal	
diverticulum		glande intestinale	

Listed in alphabetical order.

**Figure 2:**
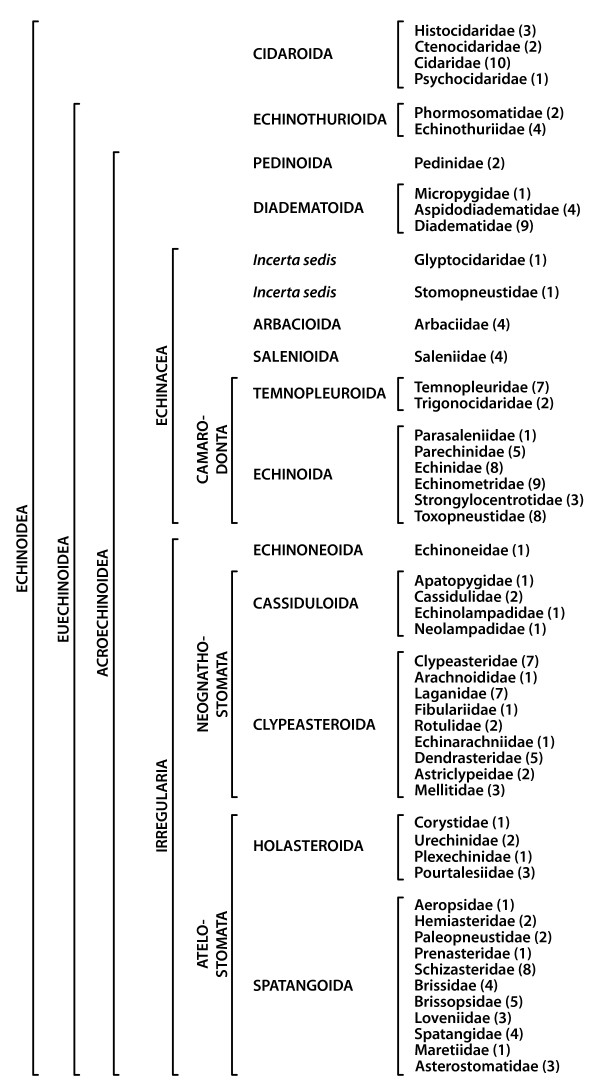
**List of higher sea urchin taxa analyzed in this study**. Note that the monophyly of several of these taxa is still under debate. The numbers in brackets designate the number of species analyzed in each family in the course of this study. This diagram is based upon results obtained by numerous authors [74-84].

In order to provide an example for the evolutionary plasticity of invertebrate digestive tract structures, we here describe the diversity observed in the morphology of the sea urchin anterior stomach by investigating taxa representing a wide diversity of forms within the Echinoidea. The aim of our study was (i) to catalogue the diversity of the anterior stomach morphology observed among sea urchins, (ii) to suggest a number of homology criteria that apply to the observed structures in all sea urchin taxa included in our analysis, (iii) to elucidate the evolutionary origin of the highly specialized gastric caecum found in the derived Spatangoida, and (iv) to evaluate implications for sea urchin phylogeny. Using magnetic resonance imaging (MRI) and three-dimensional (3D) reconstruction in combination with dissection and an extensive literature survey, we were able to incorporate 168 sea urchin species belonging to 51 extant families into our analysis (Figure 2). This comprehensive survey will serve as a basis for future studies involving the ecology, histology, ultrastructure, and function of digestive tract structures in a major taxon of the invertebrate macrozoobenthos.

## Results

The following descriptions give an overview of the anterior stomach found in 51 echinoid families (Figure 2). We focus here on the general location of this part of the digestive tract within members of each family as well as the presence or absence of sub-structures. Intra- and inter-specific variability exists for certain internal structures in sea urchins [25]. Therefore, we only mention the relevant deviations from our general findings at the family level. We regard the anterior stomach as beginning immediately distal to the junction of esophagus and stomach in the vicinity of the branching-off point of the primary siphon (some irregular taxa possess a secondary siphon [5]). A certain degree of histological specialization is known to exist in the anterior part of the stomach in "regular" taxa [24,26,27]. The primary siphon, although a derivative of the entire stomach and therefore also present in the anterior stomach, is not considered here, primarily because histological techniques not used in this study have been shown to be essential in determining presence or absence of the primary siphon [28].

**Figure 3:**
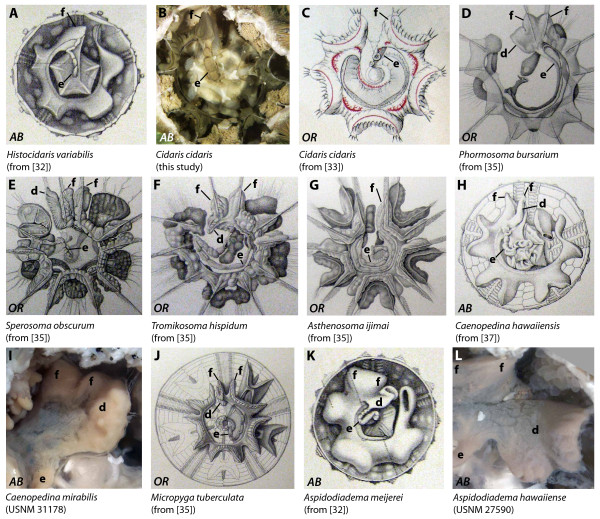
**Digestive tract anatomy of selected "regular" sea urchin taxa (Histocidaridae - Aspidodiadematidae)**. Histocidaridae (A), Cidaridae (B, C), Phormosomatidae (D), Echinothuriidae (E-G), Pedinidae (H, I), Micropygidae (J), and Aspidodiadematidae (K, L). *AB *= aboral view, *OR *= oral view. d = dilation, e = esophagus, f = festoon. Not to scale.

**Figure 4:**
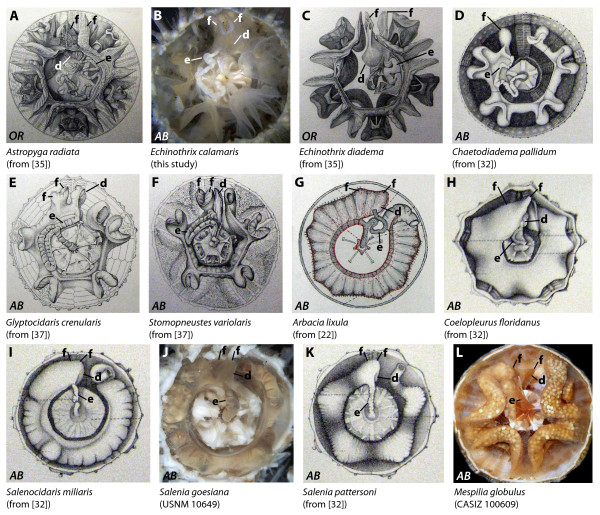
**Digestive tract anatomy of selected "regular" sea urchin taxa (Diadematidae - Temnopleuridae)**. Diadematidae (A-D), Glyptocidaridae (E), Stomopneustidae (F), Arbaciidae (G, H), Saleniidae (I-K), and Temnopleuridae (L). (G) from [22, Fig. 2, Pl. II] - reproduced in modified form with kind permission from L'Institut Océanographique, Fondation Albert Ier, Prince de Monaco. *AB *= aboral view, *LA *= lateral view, *OR *= oral view. d = dilation, e = esophagus, f = festoon. Not to scale.

**Figure 5:**
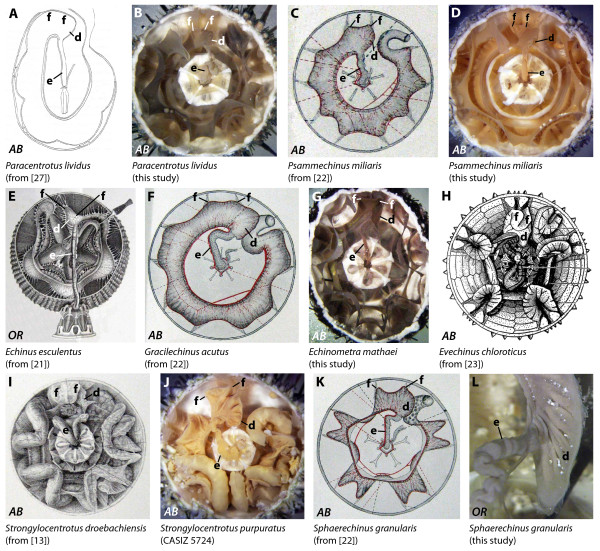
**Digestive tract anatomy of selected "regular" sea urchin taxa (Parechinidae - Toxopneustidae)**. Parechinidae (A-D), Echinidae (E, F), Echinometridae (G, H), Strongylocentrotidae (I, J), and Toxopneustidae (K, L). (A) from [27, Fig. 9] - reproduced in modified form with kind permission from Mr. Thierry Powis de Tenbossche. (C, F, K) from [22, Figs. 1, 4, 7, Pl. II] - reproduced in modified form with kind permission from L'Institut Océanographique, Fondation Albert Ier, Prince de Monaco. (H) from [23, Fig. 9] - reproduced in modified form with kind permission from The Royal Society of New Zealand. *AB *= aboral view, *OR *= oral view. d = dilation, e = esophagus, f = festoon. Not to scale.

**Figure 6:**
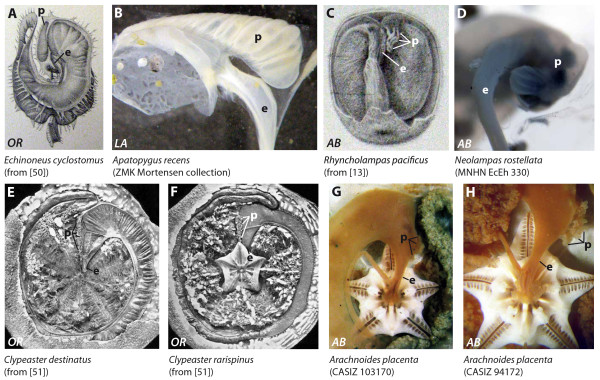
**Digestive tract anatomy of selected irregular sea urchin taxa (Echinoneidae - Arachnoididae)**. Echinoneidae (A), Apatopygidae (B), Cassidulidae (C), Neolampadidae (D), Clypeasteridae (E, F), and Arachnoididae (G = juvenile specimen, H = adult specimen). *AB *= aboral view, *LA *= lateral view, *OR *= oral view. e = esophagus, p = pouch. Not to scale.

**Figure 7:**
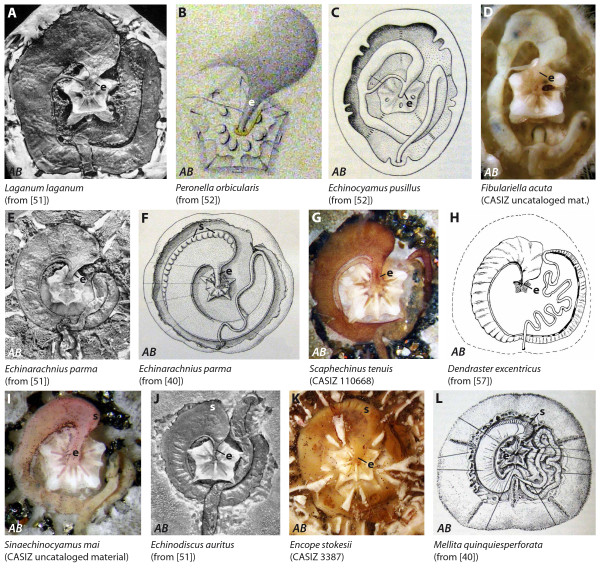
**Digestive tract anatomy of selected irregular sea urchin taxa (Laganidae - Mellitidae)**. Laganidae (A, B), Fibulariidae (C), Rotulidae (D), Echinarachniidae (E, F), Dendrasteridae (G-I), Astriclypeidae (J), and Mellitidae (K, L). (H) from [57, Fig. 3] - reproduced in modified form with kind permission from the Marine Biological Laboratory, Woods Hole, MA, USA. *AB *= aboral view. e = esophagus, s = sacculated abaxial edge of the stomach. Not to scale.

**Figure 8:**
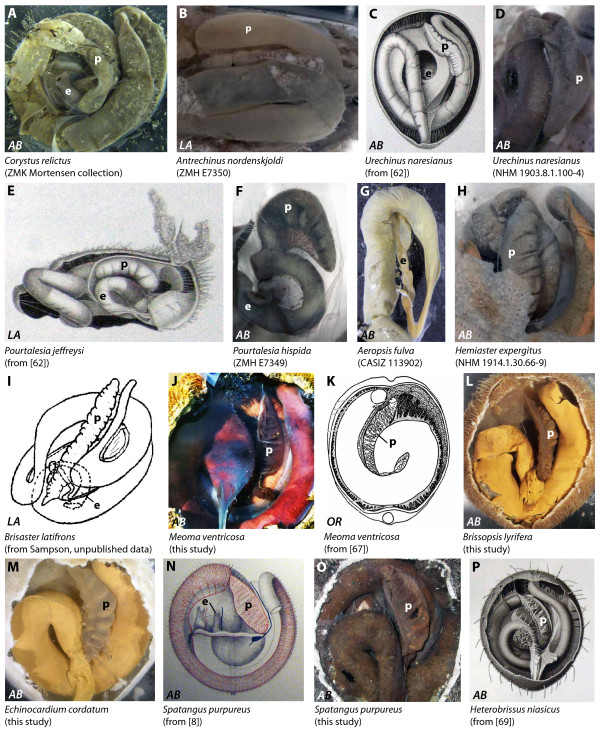
**Digestive tract anatomy of selected irregular sea urchin taxa (Corystidae - Spatangidae)**. Corystidae (A), Urechinidae (B-D), Pourtalesiidae (E, F), Aeropsidae (G), Hemiasteridae (H), Schizasteridae (I), Brissidae (J, K), Brissopsidae (L), Loveniidae (M), Spatangidae (N, O), and Asterostomatidae (P). (K) from [67, Fig. 11] - reproduced in modified form with kind permission from the Rosenstiel School of Marine and Atmospheric Science. *AB *= aboral view, *LA *= lateral view, O*R *= oral view. e = esophagus, p = pouch. Not to scale.

**Figure 9:**
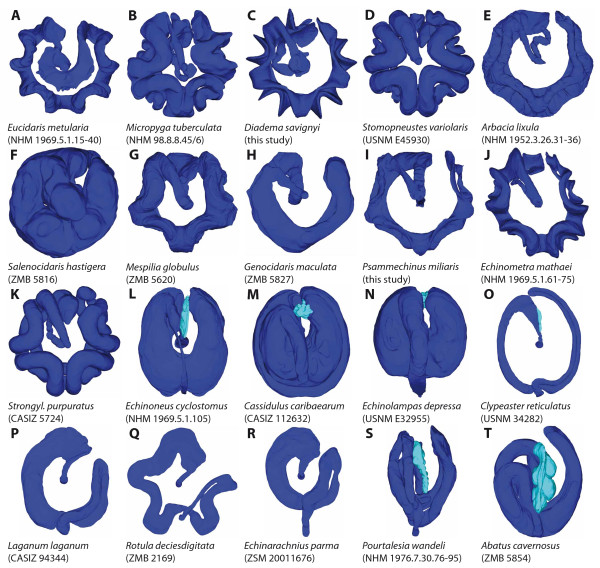
**Comparative anatomy of the sea urchin digestive tract**. (A-T) Aboral views of 3D models that were produced based on magnetic resonance imaging scans of 20 sea urchin species. Cidaridae (A), Micropygidae (B), Diadematidae (C), Stomopneustidae (D), Arbaciidae (E), Saleniidae (F), Temnopleuridae (G), Trigonocidaridae (H), Parechinidae (I), Echinometridae (J), Strongylocentrotidae (K), Echinoneidae (L), Cassidulidae (M), Echinolampadidae (N), Clypeasteridae (O), Laganidae (P), Rotulidae (Q), Echinarachniidae (R), Pourtalesiidae (S), Schizasteridae (T). Dark blue = main digestive tract (comprising the lateral dilation in "regular" euechinoid species (B-K)); cyan = thin-walled pouch(es) in irregular sea urchin species. Not to scale.

**Figure 10:**
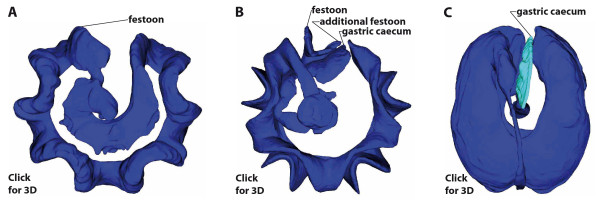
**Homology of the sea urchin gastric caecum based on its location as a primary criterion**. (A-C) Interactive 3D PDF models of the digestive tract of two "regular" [*Eucidaris metularia *(A), *Diadema savignyi *(B)] and one irregular [*Echinoneus cyclostomus *(C)] sea urchin species. Left-click onto each of the three images in order to activate the embedded 3D models. Labeling designates the structures we consider homologous. Note that the 3D model of *Diadema savignyi *(B) depicts a modelling artefact due to the close proximity of esophagus and rectum: both structures seem to be fused, although they are clearly not in reality. Please refer to [88-90] for an in-depth explanation of how to manipulate and generate publication-embedded 3D PDF models. This interactive 3D figure requires Adobe Reader 8.0 or higher to operate. Not to scale.

The topographic reference system for our descriptions is based upon Lovén's system [29] as depicted in Figure 1 A, B [ambulacra I-V (Amb I-V) and interambulacra 1-5 (IAmb 1-5)]. Furthermore, Figures 3, 4, 5, 6, 7, 8 denote whether the specimen is viewed aborally (*AB*), laterally (*LA*), or orally (*OR*). Figure 9 provides an overview of the general sea urchin digestive tract morphology - the models presented in this figure are entirely based on 3D MRI datasets [25,30]. Finally, Figure 10 provides three interactive 3D models of the digestive tracts of selected taxa. In all figures within the present article, except for a number of lateral views, Amb III is always facing upwards. The images taken from the literature have in some cases been modified slightly through removal of labels used by the original author(s). All images were chosen based on the quality and plausibility in the manner in which digestive tract structures in particular had been depicted.

Specimens were aligned according to Lovén's system by first locating the axial complex within the specimen. The axial complex is a structure formed by various primary and secondary body cavities which is located in IAmb 2 underneath the madreporic plate - see [31] for a survey of this structure within the Echinoidea. Tables 2 and 3 provide information on all species analyzed within this study, in particular on the method forming the basis of the description (i.e. dissection, MRI, or literature references [32-70]).

**Table 2:** List of "regular" sea urchin taxa included in this study.

Order	Family	Species	Method used	Specimen ID	Reference
Cidaroida Claus, 1880	Histocidaridae Lambert, 1900	*Histocidaris elegans *(Agassiz, 1879)	MRI (81 μm)^3^	ZMH E907	this study

		*Histocidaris variabilis *(Agassiz & Clark, 1907)	Dissection	-	[32]

		*Poriocidaris purpurata *(Wyville Thomson, 1872)	Dissection	-	this study

	Ctenocidaridae Mortensen, 1928	*Ctenocidaris nutrix *(Wyville Thomson, 1876)	MRI (79 μm)^3^, dissection	NHM 1956.10.5.1, AAD uncataloged material	this study

		*Notocidaris gaussensis *Mortensen, 1909	MRI (79 μm)^3^	ZMB 5456	this study

	Cidaridae Gray, 1825	*Austrocidaris canaliculata *(Agassiz, 1863)	MRI (79 μm)^3^	ZMB 2244	this study

		*Cidaris cidaris *(Linnaeus, 1758)	MRI (81 μm)^3^, dissection	NHM 1925.10.30.103-113, ZMB 4803	[33], this study

		*Eucidaris metularia *(de Lamarck, 1816)	MRI (81 μm)^3^	NHM 1969.5.1.15-40	this study

		*Eucidaris thouarsii *(Agassiz & Desor, 1847)	MRI 50 × 50 × 200 μm^3^	ZMB 1369	this study

		*Eucidaris tribuloides *Desmoulins, 1835	MRI 50 × 50 × 200 μm^3^	ZMB 5474	this study

		*Goniocidaris parasol *Fell, 1958	Dissection	NIWA 18974	this study

		*Hesperocidaris panamensis *(Agassiz, 1898)	MRI 50 × 50 × 200 μm^3^	ZMB 5407	this study

		*Phyllacanthus parvispinus *Tenison Woods, 1880	Dissection	-	this study

		*Stereocidaris indica *Döderlein, 1901	MRI (79 μm)^3^	ZMB 7364	this study

		*Stylocidaris affinis *(Philippi, 1845)	Dissection	-	this study

	Psychocidaridae Ikeda, 1936	*Psychocidaris ohshimai *Ikeda, 1935	MRI (79 μm)^3^	NHMW 200Z0097/0001	this study

Echinothurioida Claus, 1880	Phormosomatidae Mortensen, 1934	*Phormosoma bursarium *Agassiz, 1881	Dissection	NIWA 45056, AM J.16209	[34,35], this study

		*Phormosoma placenta *Wyville Thomson, 1872	Dissection	ZMK Mortensen collection	this study

	Echinothuriidae Wyville Thomson, 1872	*Asthenosoma ijimai *Yoshiwara, 1897	Dissection	-	[35]

		*Sperosoma obscurum *Agassiz & Clark, 1907	Dissection	-	[35]

		*Tromikosoma hispidum *(Agassiz, 1898)	Dissection	-	[35]

		*Tromikosoma tenue *(Agassiz, 1879)	Dissection	-	[36]

Pedinoida Mortensen, 1939	Pedinidae Pomel, 1883	*Caenopedina hawaiiensis *Agassiz & Clark, 1907	Dissection	-	[37]

		*Caenopedina mirabilis *(Döderlein, 1885)	MRI (81 μm)^3^, dissection	USNM 31178, USNM 31182, AM J.24188	this study

Diadematoida Duncan, 1889	Micropygidae Mortensen, 1903	*Micropyga tuberculata *Agassiz, 1879	MRI (81 μm)^3^, dissection	NHM 98.8.8.45/6, ZMK Mortensen collection	[35,38], this study

	Aspidodiadematidae Duncan, 1889	*Aspidodiadema hawaiiense *Mortensen, 1939	MRI (81 μm)^3^, dissection	USNM 27590	this study

		*Aspidodiadema jacobi *Agassiz, 1880	Dissection	-	[39]

		*Aspidodiadema meijerei *(Döderlein, 1906)	Dissection	-	[32]

		*Plesiodiadema indicum *(Döderlein, 1900)	MRI (81 μm)^3^	ZMB 7232	this study

	Diadematidae Gray, 1855	*Astropyga radiata *(Leske, 1778)	Dissection	-	[35]

		*Centrostephanus longispinus *(Philippi, 1845)	MRI (66 μm)^3^	NHM 1952.3.26.64-8	this study

		*Centrostephanus rodgersii *(Agassiz, 1863)	Dissection	-	this study

		*Chaetodiadema pallidum *(Agassiz & Clark, 1907)	Dissection	-	[32]

		*Diadema antillarum *Philippi, 1845	MRI 50 × 50 × 200 μm^3^, dissection	ZMB 4374	[24], this study

		*Diadema savignyi *Michelin, 1845	MRI (40 μm)^3^	-	this study

		*Diadema setosum *(Leske, 1778)	Dissection	-	[13,28], this study

		*Echinothrix calamaris *(Pallas, 1774)	Dissection	-	this study

		*Echinothrix diadema *(Linnaeus, 1758)	MRI 50 × 50 × 200 μm^3^, dissection	ZMB 2346	[35], this study

*Incerta sedis*	Glyptocidaridae Jensen, 1982	*Glyptocidaris crenularis *Agassiz, 1864	Dissection	-	[37]

*Incerta sedis*	Stomopneustidae Mortensen, 1903	*Stomopneustes variolaris *(de Lamarck, 1816)	MRI (81 μm)^3^, dissection	USNM E45930	[37], this study

Arbacioida Gregory, 1900	Arbaciidae Gray, 1855	*Arbacia dufresnii *(de Blainville, 1825)	MRI 50 × 50 × 200 μm^3^	ZMB 2222	this study

		*Arbacia lixula *(Linnaeus, 1758)	MRI (81 μm)^3^, dissection	NHM 1952.3.26.31-36, ZMB 7203	[22], this study

		*Arbacia punctulata *(de Lamarck, 1816)	Dissection	-	[40]

		*Coelopleurus floridanus *Agassiz, 1871	Dissection	-	[32]

Salenioida Delage & Herouard, 1903	Saleniidae Agassiz, 1838	*Salenia goesiana *Lovén, 1874	Dissection	USNM 10649	this study

		*Salenia pattersoni *Agassiz, 1878	Dissection	-	[32]

		*Salenocidaris hastigera *Agassiz, 1869	MRI (81 μm)^3^	ZMB 5816	this study

		*Salenocidaris miliaris *Agassiz, 1869	Dissection	-	[32]

Temnopleuroida Mortensen, 1942	Temnopleuridae Agassiz, 1872	*Amblypneustes pallidus *(de Lamarck, 1816)	MRI 50 × 50 × 200 μm^3^	ZMB 6334	this study

		*Holopneustes inflatus *Lutken, 1872	MRI 50 × 50 × 200 μm^3^	ZMB 2639	this study

		*Mespilia globulus *(Linnaeus, 1758)	MRI (44 μm)^3^	ZMB 5620, CASIZ 100609	this study

		*Pseudechinus magellanicus *Philippi, 1857	MRI 50 × 50 × 200 μm^3^	ZMB 2188	this study

		*Salmacis bicolor *(Agassiz, 1846)	Dissection	-	[41]

		*Temnopleurus michaelseni *(Döderlein, 1914)	MRI 50 × 50 × 200 μm^3^	ZMB 6331	this study

		*Temnopleurus toreumaticus *(Leske, 1778)	MRI 78 × 78 × 300 μm^3^, dissection	ZMB 5511, ZMB 2802	this study

	Trigonocidaridae Mortensen, 1903	*Genocidaris maculata *Agassiz, 1869	MRI (36 μm)^3^	ZMB 5827	this study

		*Trigonocidaris albida *Agassiz, 1869	MRI (32 μm)^3^	ZSM 20012468	this study

Echinoida Troschel, 1872	Parasaleniidae Mortensen, 1940	*Parasalenia gratiosa *Agassiz, 1864	MRI (79 μm)^3^	NHM 1983.2.15.7	this study

	Parechinidae Mortensen, 1903	*Loxechinus albus *(Molina, 1782)	MRI 50 × 50 × 200 μm^3^	NHM 1966.9.27.35	this study

		*Paracentrotus lividus *(de Lamarck, 1816)	MRI (81 μm)^3^, dissection	-	[20-22,27], this study

		*Parechinus angulosus *(Leske, 1778)	MRI 50 × 50 × 200 μm^3^	ZMB 5644	this study

		*Psammechinus microtuberculatus *(de Blainville, 1825)	MRI 50 × 50 × 200 μm^3^, dissection	ZMB 4770	[22], this study

		*Psammechinus miliaris *(Müller, 1771)	MRI (44 μm)^3^, dissection	-	[22,42], this study

	Echinidae Gray, 1825	*Echinus esculentus *Linnaeus, 1758	MRI (81 μm)^3^, dissection	ZMB 3826	[21,43-46], this study

		*Echinus melo *de Lamarck, 1816	Dissection	-	[9]

		*Gracilechinus acutus *(de Lamarck, 1816)	MRI 78 × 78 × 300 μm^3^, dissection	ZMB 3604	[22], this study

		*Gracilechinus alexandri *(Danielssen & Koren, 1883)	MRI 50 × 50 × 200 μm^3^	ZMB 4340	this study

		*Polyechinus agulhensis *(Döderlein, 1905)	MRI 50 × 50 × 200 μm^3^	ZMB 7219	this study

		*Sterechinus agassizi *Mortensen, 1910	MRI (79 μm)^3^	NHM 1914.8.12.126-127	this study

		*Sterechinus antarcticus *Koehler, 1901	MRI 50 × 50 × 200 μm^3^	ZMB 5439	this study

		*Sterechinus neumayeri *(Meissner, 1900)	MRI 50 × 50 × 200 μm^3^	ZMB uncataloged material	this study

	Echinometridae Gray, 1855	*Caenocentrotus gibbosus *(Agassiz & Desor, 1840)	MRI 50 × 50 × 200 μm^3^	ZMB 5405	this study

		*Echinometra lucunter *(Linnaeus, 1758)	Dissection	ZMB 5511	this study

		*Echinometra mathaei *(de Blainville, 1825)	MRI (81 μm)^3^	NHM 1969.5.1.61-75	this study

		*Echinometra viridis *Agassiz, 1863	MRI 50 × 50 × 200 μm^3^, dissection	ZMB 1827, ZMB 5503	this study

		*Echinostrephus molaris *(de Blainville, 1825)	MRI 50 × 50 × 200 μm^3^	ZMB 4000	this study

		*Evechinus chloroticus *(Valenciennes, 1846)	Dissection	-	[23]

		*Heliocidaris crassispina *(Agassiz, 1863)	MRI 50 × 50 × 200 μm^3^	ZMB 6424	this study

		*Heliocidaris erythrogramma *(Valenciennes, 1846)	MRI 50 × 50 × 200 μm^3^	ZMB 5745	this study

		*Heterocentrotus mammillatus *(Linnaeus, 1758)	MRI 50 × 50 × 200 μm^3^	ZMB 1567	this study

	Strongylocentrotidae Gregory, 1900	*Pseudocentrotus depressus *(Agassiz, 1863)	MRI 50 × 50 × 200 μm^3^	ZMB 6426	this study

		*Strongylocentrotus droebachiensis *(Müller, 1776)	MRI 50 × 50 × 200 μm^3^, dissection	ZMB 4446, ZMB 4422	[13,47], this study

		*Strongylocentrotus purpuratus *(Stimpson, 1857)	MRI (44 μm)^3^, dissection	CASIZ 5724	[26], this study

	Toxopneustidae Troschel, 1872	*Gymnechinus robillardi *(de Loriol, 1883)	MRI (79 μm)^3^	NHM 1890.6.27.5-8	this study

		*Lytechinus variegatus *(de Lamarck, 1816)	MRI 50 × 50 × 200 μm^3^, dissection	ZMB 5517	this study

		*Nudechinus scotiopremnus *Clark, 1912	MRI 50 × 50 × 200 μm^3^	ZMB 6130	this study

		*Sphaerechinus granularis *(de Lamarck, 1816)	MRI (81 μm)^3^, dissection	ZMB 2366, ZMB 7204	[22,48], this study

		*Toxopneustes pileolus *(de Lamarck, 1816)	MRI 50 × 50 × 200 μm^3^	ZMB 3871	this study

		*Tripneustes esculentus *(Leske, 1778)	MRI 50 × 50 × 200 μm^3^	ZMB 5498	this study

		*Tripneustes gratilla *(Linnaeus, 1758)	MRI 78 × 78 × 300 μm^3^	ZMB 3863	this study

		*Tripneustes ventricosus *de Lamarck, 1816	Dissection	-	[49]


The table provides information on every species studied so far with regard to digestive tract anatomy, listing the method(s) used, the specimen ID of museum specimens where applicable, and the respective references. Numbers in brackets behind "MRI" represent the resolution of the dataset. An overview of scanning parameters is provided by [25,86]. this study = specimens were dissected and/or scanned in the course of this study; see the 'List of abbreviations used' section for an explanation of abbreviations.

### "Regularia"

The digestive tract of "regular" sea urchin species consists of two loops that lie more or less on top of each other and usually bear so-called festoons, i.e. vertical inflections of the gut (Figure 9A-K).

#### Histocidaridae

The anterior stomach of *Histocidaris elegans*, *Histocidaris variabilis *(Figure 3A), and *Poriocidaris purpurata *is located in Amb III. The slightly curving esophagus is initially directed towards Amb III. The anterior stomach spans Amb III horizontally and is composed of a single festoon. A small dilation extends adapically immediately distal to the junction of esophagus and stomach.

#### Ctenocidaridae

The morphology of the anterior stomach in *Ctenocidaris nutrix *and *Notocidaris gaussensis *closely resembles that found in the Histocidaridae. The anterior stomach spans Amb III horizontally close to its connection with the esophagus, and is composed of a single festoon. A small dilation extends adapically immediately distal to the junction of esophagus and stomach.

#### Cidaridae

*Austrocidaris canaliculata, Cidaris cidaris *(Figure 3B, C), *Eucidaris metularia *(Figure 9A, interactive Figure 10A), *Eucidaris thouarsii, Eucidaris tribuloides, Goniocidaris parasol*, *Hesperocidaris panamensis, Phyllacanthus parvispinus*, and *Stylocidaris affinis *are characterized by an anterior stomach that is located at the border of Amb III and IAmb 3. The esophagus is initially directed towards IAmb 3-Amb III and consists of a short, straight tube. The anterior stomach consists of a single festoon. A small dilation extends adapically just distal to the junction of esophagus and stomach. In his detailed description of the internal anatomy of *Cidaris cidaris*, Prouho [33] specifically mentioned the absence of any kind of caecum at the junction of esophagus and stomach. *Stereocidaris indica *deviates from this general description, with a situation that more closely resembles that found in the Histocidaridae and the Ctenocidaridae.

#### Psychocidaridae

The anterior stomach of *Psychocidaris ohshimai *is located at the border of IAmb 3 and Amb III. The esophagus is initially directed towards Amb III and consists of a short, slightly curved tube. The anterior stomach consists of a single festoon. A small dilation extends adapically and towards IAmb 2 just distal to the junction of esophagus and stomach.

#### Phormosomatidae

A long esophagus that is initially directed towards Amb I connects the pharynx to the anterior stomach in *Phormosoma bursarium *(Figure 3D) and *Phormosoma placenta. *The anterior stomach spans Amb III and is located between IAmb 3 and IAmb 2. It consists of two separate festoons and a large lateral dilation within IAmb 2. Unfortunately, Schurig [34] did not specifically mention the morphology of the anterior stomach in his detailed report on the internal anatomy of *Phormosoma bursarium *and a number of other species of the Echinothurioida.

#### Echinothuriidae

The digestive tract of the echinothuriid species analyzed so far is characterized by a long esophagus which can sometimes double back on itself. The esophagus in *Sperosoma obscurum *(Figure 3E) is initially directed towards Amb II but connects with the anterior stomach in Amb III after making almost a full turn. The anterior stomach of this species consists of two large, separate festoons and a large dilation in IAmb 2. The size of the dilation can vary in echinothuriid species, being largest in *Sperosoma obscurum *and rather medium-sized in *Tromikosoma hispidum *(Figure 3F) and *Tromikosoma tenue*. The anterior stomach of *Asthenosoma ijimai *(Figure 3G) differs in lacking the dilation as well as the additional festoon of the anterior stomach - however, a conspicuous structure, drawn as part of the intestine, occupies the respective void in Amb III.

#### Pedinidae

The species of the genus *Caenopedina *that have been analyzed so far possess an anterior stomach located between IAmb 3 and Amb III. The winding esophagus is initially directed towards Amb IV in *Caenopedina hawaiiensis *(Figure 3H) and *Caenopedina mirabilis *(Figure 3I). The anterior stomach consists of two horizontally fused festoons and a lateral dilation located in Amb III.

#### Micropygidae

The anterior stomach of *Micropyga tuberculata *(Figures 3J, 9B) is located in Amb III, but also reaches laterally into IAmb 2. The winding esophagus is initially directed towards IAmb 5, but connects to the anterior stomach in Amb III. The anterior stomach consists of two separate festoons and a lateral dilation located in IAmb 2. This dilation extends considerably along the oral-aboral axis of the species. Mortensen [38: 142] also noted that "at the passage from the long oesophagus to the intestine there is a large blindsac" (Mortensen's usage of "intestine" = stomach in the present article).

#### Aspidodiadematidae

The anterior stomach of *Aspidodiadema jacobi, Aspidodiadema meijerei *(Figure 3K), *Aspidodiadema hawaiiense *(Figure 3L), and *Plesiodiadema indicum *is located between IAmb 3 and IAmb 2. The winding esophagus is initially directed towards Amb IV before connecting to the anterior stomach in Amb III. The anterior stomach consists of two separate festoons and a conspicuous lateral dilation located in IAmb 2.

#### Diadematidae

Diadematids possess a complex anterior stomach that is located between IAmb 2 and IAmb 3. The winding esophagus is initially directed towards Amb IV or V. The anterior part of the stomach in *Astropyga radiata *(Figure 4A), *Diadema antillarum*, *Diadema savignyi *(Figure 9C, interactive Figure 10B), *Diadema setosum*, *Echinothrix calamaris *(Figure 4B), and *Echinothrix diadema *(Figure 4C) consists of two separate festoons and a well-developed dilation. This dilation seems to be less developed in *Centrostephanus longispinus *and *Centrostephanus rodgersii*. However, in *Chaetodiadema pallidum *(Figure 4D), the anterior stomach is characterized by a single festoon only which is located in IAmb 3. Both the additional festoon and the dilation are absent from this species. According to Lewis [24: 552], the digestive tract of *Diadema antillarum *consisted "of five sections: esophagus, caecum, foregut, hindgut, and rectum" and "The caecum is a large blind sac. It is continuous with the first loop of the foregut but can be distinguished from the latter by its brighter colour". Lewis also mentioned the presence of "a valve at the junction of caecum and foregut". The above-mentioned dilation can be seen in a recent picture of *Diadema setosum *(Figure 1A in 28: note the adaxial part of the crenulated structure at the right hand side of the junction of esophagus (es) and stomach (st)). In some diadematid species, particularly in *Astropyga*, *Diadema*, and *Echinothrix*, a fusion of both "ends" of the lower gut loop through mesenteries can be observed at the border of Amb III and IAmb 2 (Figure 4A).

#### Glyptocidaridae

The only extant species in this family, *Glyptocidaris crenularis *(Figure 4E), is characterized by the presence of an anterior stomach that is located in Amb III. The winding esophagus is initially directed towards Amb IV. The anterior stomach consists of two separate festoons and a large dilation that extends slightly into IAmb 2.

#### Stomopneustidae

The digestive tract of *Stomopneustes variolaris *(Figures 4F, 9D) is very similar to that found in *Glyptocidaris crenularis*. The anterior stomach consists of two separate festoons. The esophagus displays some degree of morphological variation and may initially be directed towards Amb III or Amb IV. A large dilation is present at the border of Amb III and IAmb 2.

#### Arbaciidae

The arbaciid species analyzed so far, *Arbacia dufresnii, Arbacia lixula *(Figures 4G, 9E), *Arbacia punctulata*, and *Coelopleurus floridanus *(Figure 4H), possess an anterior stomach that is located in Amb III. The esophagus is initially directed towards Amb III, but later undulates towards IAmb 2 proximal to its junction with the stomach in Amb III. Due to the horizontally more depressed aspect of the entire arbaciid gut, differentiation of separate festoons and dilations is difficult. However, slight undulations of mesenteries indicate the presence of two horizontally fused festoons in Amb III as well as a small dilation extending into IAmb 2.

#### Saleniidae

Similar to that in arbaciid species, the saleniid gut does not exhibit pronounced vertical festooning. In *Salenocidaris miliaris *(Figure 4I), *Salenia goesiana *(Figure 4J), *Salenocidaris hastigera *(Figure 9F), and *Salenia pattersoni *(Figure 4K), the short esophagus is initially directed towards Amb III where it connects to the anterior stomach. This part of the gut spans between Amb III and IAmb 3 and displays a small lateral dilation directed towards IAmb 2. Slight undulations of mesenteries indicate the presence of two horizontally fused festoons in Amb III.

#### Temnopleuridae

Information on the internal anatomy of temnopleurids is available for *Amblypneustes pallidus*, *Holopneustes inflatus*, *Mespilia globulus *(Figures 4L, 9G)*, Pseudechinus magellanicus, Salmacis bicolor*, *Temnopleurus michaelseni*, and *Temnopleurus toreumaticus*. MRI analyses indicate that in all species the slightly winding esophagus is initially directed towards Amb III. The anterior stomach is characterized by the presence of two horizontally fused festoons located in Amb III. A small dilation is directed adapically and towards IAmb 2.

#### Trigonocidaridae

MRI scans of *Genocidaris maculata *(Figure 9H) and *Trigonocidaris albida *reveal the presence of the anterior stomach in Amb III. The short esophagus is initially directed towards Amb III, where it connects to the stomach. Whereas the anterior stomach in *Trigonocidaris albida *spans the entire Amb III, also reaching into IAmb 2, the anterior stomach in *Genocidaris maculata *is located mainly in IAmb 3 and only slightly extends into Amb III. However, both species are characterized by a small dilation of the anterior stomach pointing adapically distal to the junction of esophagus and stomach. Due to the flattened aspect of the entire stomach it is hard to differentiate individual festoons, but slight undulations of mesenteries indicate the presence of two horizontally fused festoons in Amb III.

#### Parasaleniidae

All known species in this monogeneric family are characterized by an oval test. In *Parasalenia gratiosa*, the only species analyzed here, the anterior stomach is largely located in Amb III, but reaches into IAmb 2 and 3 as well. The winding esophagus is initially directed towards IAmb 3, but distally bends towards Amb III where it enters the stomach. The anterior stomach consists of two separate festoons as well as a large dilation located at the border of Amb III and IAmb 2. This dilation extends towards the apex.

#### Parechinidae

In specimens of *Loxechinus albus, Paracentrotus lividus *(Figures 1B, D; 5A, B), *Parechinus angulosus, Psammechinus microtuberculatus*, and *Psammechinus miliaris *(Figures 5C, D; 9I), the anterior stomach is located in Amb III. The short esophagus is initially directed towards Amb III where it also connects to the anterior stomach. The two individual festoons in Amb III are horizontally fused. A small dilation is located just distal to the junction of esophagus and anterior stomach. Several authors have noted this dilation in *Paracentrotus lividus*, among them Koehler [9], Tiedemann [20], Valentin [21], and Bonnet [22].

#### Echinidae

Data on the anterior stomach of echinids are available for eight species: *Echinus esculentus *(Figure 5E), *Echinus melo*, *Gracilechinus acutus *(Figure 5F), *Gracilechinus alexandri*, *Polyechinus agulhensis*, *Sterechinus agassizi*, *Sterechinus antarcticus*, and *Sterechinus neumayeri*. In all species, the anterior stomach is largely located in Amb III, but also extends slightly into IAmb 2. The moderately winding esophagus is initially directed towards Amb III, where it also connects to the anterior stomach. This part of the digestive tract is characterized by the presence of two horizontally fused festoons as well as a small dilation that reaches into IAmb 2. According to Koehler [9], this dilation distal to the junction of the esophagus with the anterior stomach is more developed in *Echinus melo *than in *Echinus esculentus*.

#### Echinometridae

This taxon incorporates species with either an oval or circular test. The anterior stomach in *Caenocentrotus gibbosus*, *Echinometra lucunter*, *Echinometra mathaei *(Figures 5G, 9J), *Echinometra viridis*, *Echinostrephus molaris*, *Evechinus chloroticus *(Figure 5H), *Heliocidaris crassispina, Heliocidaris erythrogramma*, and *Heterocentrotus mammillatus *is largely located in Amb III, but extends into IAmb 2 and 3 as well. The winding esophagus is initially directed towards the apex before descending towards IAmb 3; it joins the anterior stomach in Amb III. The anterior stomach is characterized by the presence of two separate festoons and a large, adapically oriented dilation located near IAmb 2. McRae [23: 238] described the internal anatomy of *Evechinus chloroticus *and noted that the anterior stomach is characterized by the presence of a "sac-like dilatation" that is connected to esophagus and axial complex "through sheets of mesentery".

#### Strongylocentrotidae

The anterior stomach of *Pseudocentrotus depressus*, *Strongylocentrotus droebachiensis *(Figure 5I), and *Strongylocentrotus purpuratus *(Figures 5J; 9K) is located in Amb III. The moderately winding esophagus is initially directed towards the apex before descending towards IAmb 3; it later connects to the anterior stomach in Amb III. The two individual festoons in Amb III are horizontally fused for parts of their length. A small dilation occurs immediately distal to the connection of esophagus and stomach. This dilation is directed adapically and extends into IAmb 2.

#### Toxopneustidae

The species that so far have been analyzed in this taxon all possess a prominent anterior stomach located in Amb III. In *Gymnechinus robillardi, Lytechinus variegatus*, *Nudechinus scotiopremnus, Sphaerechinus granularis *(Figure 5K, L), *Toxopneustes pileolus, Tripneustes esculentus, Tripneustes gratilla*, and *Tripneustes ventricosus*, the two individual festoons of the anterior stomach are moderately fused and located in Amb III. The winding, short esophagus is initially directed towards the apex before descending towards IAmb 3; it later connects to the anterior stomach in Amb III. A variably sized dilation of the anterior stomach, largely located in IAmb 2, is oriented adapically. The esophagus in *Tripneustes *spp. doubles back on itself [49].

**Table 3:** List of irregular sea urchin taxa included in this study.

Order	Family	Species	Method used	Specimen ID	Reference
Echinoneoida Clark, 1925	Echinoneidae Agassiz & Desor, 1847	*Echinoneus cyclostomus *Leske, 1778	MRI (66 μm)^3^, dissection	NHM 1969.5.1.105, ZMB 4963	[50], this study

Cassiduloida Agassiz & Desor, 1847	Apatopygidae Kier, 1962	*Apatopygus recens *(Mortensen, 1948)	Dissection	ZMK Mortensen collection	this study

	Cassidulidae Agassiz & Desor, 1847	*Cassidulus caribaearum *de Lamarck, 1801	MRI (81 μm)^3^	CASIZ 112632	[70], this study

		*Rhyncholampas pacificus *(Agassiz, 1863)	Dissection	-	[13]

	Echinolampadidae Gray, 1851	*Echinolampas depressa *Gray, 1851	MRI (81 μm)^3^	USNM E32955	this study

	Neolampadidae Lambert, 1918	*Neolampas rostellata *Agassiz, 1869	MRI (81 μm)^3^, dissection	MNHN EcEh 330	this study

Clypeasteroida Agassiz, 1835	Clypeasteridae Agassiz, 1835	*Clypeaster annandalei *Koehler, 1922	Dissection	-	[51]

		*Clypeaster destinatus *Koehler, 1922	Dissection	-	[51]

		*Clypeaster europacificus *Clark, 1914	Dissection	CASIZ 101408	this study

		*Clypeaster humilis *(Leske, 1778)	Dissection	-	[51]

		*Clypeaster rarispinus *de Meijere, 1903	Dissection	-	[51]

		*Clypeaster reticulatus *(Linnaeus, 1758)	MRI (81 μm)^3^	USNM 34282	[51], this study

		*Clypeaster rosaceus *(Linnaeus, 1758)	MRI (96 μm)^3^, dissection	ZMB 2520	[13], this study

	Arachnoididae Duncan, 1889	*Arachnoides placenta *(Linnaeus, 1758)	MRI (81 μm)^3^, dissection	ZMB 1439, CASIZ 93620, CASIZ 103170, CASIZ 94172	[51], this study

	Laganidae Agassiz, 1873	*Laganum bonani *Klein, 1734	Dissection	-	[51]

		*Laganum decagonale *de Blainville, 1827	Dissection	-	[51]

		*Laganum depressum *Agassiz, 1841	MRI (86 μm) ^3^, dissection	NHM 1932.4.28.227-34	[51], this study

		*Laganum joubini *(Koehler, 1922)	MRI (44 μm) ^3^	NHM 1979.1.25.52-60	this study

		*Laganum laganum *(Leske, 1778)	MRI (81 μm)^3^	CASIZ 94344	[51], this study

		*Peronella lesueuri *(Agassiz, 1841)	MRI (81 μm)^3^, dissection	MNHN EcEh 79	[51], this study

		*Peronella orbicularis *Leske, 1778	MRI (81 μm)^3^, dissection	MNHN EcEh 77	[52], this study

	Fibulariidae Gray, 1855	*Echinocyamus pusillus *(Müller, 1776)	MRI 20 × 18 × 18 μm^3^	-	[52], this study

	Rotulidae Gray, 1855	*Fibulariella acuta *(Yoshiwara, 1898)	Dissection	CASIZ uncataloged material	this study

		*Rotula deciesdigitata *(Leske, 1778)	MRI (81 μm)^3^, dissection	ZMB 2169	[53], this study

	Echinarachniidae Lambert, 1914	*Echinarachnius parma *(de Lamarck, 1816)	Dissection	ZSM 20011676, CASIZ 157683	[40,45,51,54], this study

	Dendrasteridae Lambert, 1900	*Dendraster excentricus *(Eschscholtz, 1831)	Dissection	-	[54-58], this study

		*Scaphechinus griseus *(Mortensen, 1927)	Dissection	-	[59]

		*Scaphechinus mirabilis *Agassiz, 1863	Dissection	-	[59]

		*Scaphechinus tenuis *(Yoshiwara, 1898)	Dissection	CASIZ 110668	this study

		*Sinaechinocyamus mai *(Wang, 1984)	Dissection	CASIZ uncatalogued material	this study

	Astriclypeidae Stefanini, 1912	*Astriclypeus mannii *Verrill, 1867	Dissection	-	this study

		*Echinodiscus auritus *Leske, 1778	Dissection	-	[51]

	Mellitidae Stefanini, 1912	*Encope stokesii *Agassiz, 1841	Dissection	CASIZ 3387	this study

		*Leodia sexiesperforata *(Leske, 1778)	Dissection	-	[59]

		*Mellita quinquiesperforata *(Leske, 1778)	Dissection	-	[40,54,60,61], this study

Holasteroida Durham & Melville, 1957	Corystidae Foster & Philip, 1978	*Corystus relictus *(de Meijere, 1902)	Dissection	ZMK Mortensen collection	this study

	Urechinidae Duncan, 1889	*Antrechinus nordenskjoldi *(Mortensen, 1905)	Dissection	ZMH E7350	this study

		*Urechinus naresianus *Agassiz, 1879	Dissection	NHM 1903.8.1.100-104	[28,62], this study

	Plexechinidae Mooi & David, 1996	*Plexechinus aoteanus *McKnight, 1974	Dissection	ZMH E7345	this study

	Pourtalesiidae Agassiz, 1881	*Pourtalesia hispida *Agassiz, 1879	Dissection	ZMH E7349	this study

		*Pourtalesia jeffreysi *Wyville Thomson, 1872	MRI (81 μm)^3^, dissection	ZSM 20011456	[62], this study

		*Pourtalesia wandeli *Mortensen, 1905	MRI (86 μm)^3^	NHM 1976.7.30.76-95	this study

Spatangoida Agassiz, 1840	Aeropsidae Lambert, 1896	*Aeropsis fulva *(Agassiz, 1881)	Dissection	CASIZ 113902	this study

	Hemiasteridae Clark, 1917	*Hemiaster expergitus *(Lovén, 1874)	Dissection	NHM 1914.1.30.66-9	this study

		*Hemiaster hickmanni *Koehler, 1914	Dissection	-	[63]

	Paleopneustidae Agassiz, 1904	*Paleopneustes cristatus *Agassiz, 1873	Dissection	-	[64]

		*Paleopneustes tholoformis *Chesher, 1968	Dissection	-	[64]

	Prenasteridae Lambert, 1905	*Prenaster enodatus *(Chesher, 1968)	Dissection	-	[64]

	Schizasteridae Lambert, 1905	*Abatus cavernosus *(Philippi, 1845)	MRI (81 μm)^3^	ZMB 5854	this study

		*Abatus cordatus *(Verrill, 1876)	Dissection	ZMB 5437	this study

		*Aceste ovata *Agassiz & Clark, 1907	Dissection	-	[63]

		*Brisaster antarcticus *(Döderlein, 1906)	Dissection	AAD uncataloged material	this study

		*Brisaster fragilis *(Duben & Koren, 1846)	Dissection	ZMB 2766	this study

		*Brisaster latifrons *(Agassiz, 1898)	Dissection	-	Sampson (unpublished data)

		*Hypselaster kempi *(Koehler, 1914)	Dissection	-	[63]

		*Schizaster canaliferus *(de Lamarck, 1816)	Dissection	-	[9,65]

	Brissidae Gray, 1855	*Brissus agassizii *Döderlein, 1885	Dissection	-	this study

		*Brissus unicolor *(Leske, 1778)	Dissection	-	[9,66]

		*Meoma ventricosa *(Lamarck, 1816)	Dissection	-	[67], this study

		*Metalia sternalis *Lamarck, 1816	Dissection	-	[13]

	Brissopsidae Lambert, 1905	*Brissopsis alta *Mortensen, 1907	Dissection	-	[64]

		*Brissopsis atlantica *Mortensen, 1907	Dissection	-	[64]

		*Brissopsis elongata *Mortensen, 1907	Dissection	-	[64]

		*Brissopsis lyrifera *(Forbes, 1841)	Dissection	ZMB 7259	[9,64], this study

		*Brissopsis mediterranea *Mortensen, 1913	Dissection	-	[64]

	Loveniidae Lambert, 1905	*Echinocardium cordatum *(Pennant, 1777)	MRI (81 μm)^3^, dissection	-	[12,15,68], this study

		*Echinocardium flavescens *(Müller, 1776)	Dissection	-	[9]

		*Lovenia subcarinata *(Gray, 1845)	Dissection	-	[4]

	Spatangidae Gray, 1825	*Plethotaenia angularis *Chesher, 1968	Dissection	-	[64]

		*Plethotaenia spatangoides *Agassiz, 1883	Dissection	-	[64]

		*Pseudomaretia alta *(Agassiz, 1863)	MRI (81 μm)^3^	ZSM 20011608	this study

		*Spatangus purpureus *Müller, 1776	MRI (81 μm)^3^, dissection	ZMB 3236	[8,9,17,66], this study

	Maretiidae Lambert, 1905	*Maretia planulata *(de Lamarck, 1816)	Dissection	-	[4]

	Asterostomatidae Pictet, 1857	*Elipneustes denudatus *(Koehler, 1914)	Dissection	-	[63]

		*Heterobrissus hemingi *(Anderson, 1902)	Dissection	-	[63]

		*Heterobrissus niasicus *(Döderlein, 1901)	Dissection	-	[69]

The table provides information on every species studied so far with regard to digestive tract anatomy, listing the method(s) used, the specimen ID of museum specimens where applicable, and the respective references. Numbers in brackets behind "MRI" represent the resolution of the dataset. An overview of scanning parameters is provided by [25,86]. this study = specimens were dissected and/or scanned in the course of this study; see the 'List of abbreviations used' section for an explanation of abbreviations.

### Irregularia

Irregular sea urchins can be distinguished from "regular" species by the absence of festoons in their entire digestive tract (Figure 9L-T).

#### Echinoneidae

The anterior stomach of *Echinoneus cyclostomus *(Figures 6A, 9L, interactive Figure 10C) is located in Amb III and also extends into IAmb 3. The short esophagus ascends from the mouth towards Amb III where it joins the anterior stomach. A large pouch is located in Amb III distal to the junction of esophagus and anterior stomach. The pouch is connected to the anterior stomach through a small opening in the middle of the lower rim of the pouch. In lateral view, the pouch is triangular, with one apex towards the echinoid's anterior edge. The aboral edge of the pouch closely follows the arched form of the test in Amb III. This pouch extends from the anterior part of the stomach in Amb III, close to the ampullae of the tube feet, until it reaches the axial complex located in the central oral-aboral axis. The surface of the pouch is covered with numerous small folds. The pouch is broader at its adoral edge than towards the apex.

#### Apatopygidae

The anterior stomach of *Apatoypgus recens *(Figure 6B) is located in Amb III. The short esophagus ascends from the mouth towards Amb III where it joins the anterior stomach. The apical side of the anterior stomach is characterized by the presence of a large pouch that extends obliquely within Amb III from the anterior part close to the test, until it reaches the axial complex that bulges anteriorly. The pouch is connected to the anterior stomach through a broad canal located at the anterior end of its adoral edge. The general form of the pouch is triangular, the anterior point being located in Amb III. The surface of the pouch is characterized by numerous folds running along the oral-aboral axis. The aboral edge of the pouch closely follows the arched form of the test in Amb III.

#### Cassidulidae

In *Cassidulus caribaearum *(Figure 9M) and *Rhyncholampas pacificus *(Figure 6C), the anterior stomach is located in Amb III and IAmb 3. The short esophagus ascends from the mouth towards Amb III until it joins the anterior stomach. At the junction of esophagus and anterior stomach, a cluster of small, smooth, finger-like pouches can be found. These pouches are directed towards the apex of Amb III and split into two smaller clusters, one directed laterally towards IAmb 2, the other oriented towards IAmb 3. The number of pouches in an adult specimen of *Cassidulus caribaearum *was found to be approximately four to six on each side. The apical side of the entire structure is in close proximity to the apical part of the test of Amb III. Agassiz [13] and Gladfelter [70] provided similar descriptions of this cluster of pouches located at the apex of the anterior stomach.

#### Echinolampadidae

The anterior stomach of *Echinolampas depressa *(Figure 9N) is primarily located in IAmb 3, but reaches into Amb III as well. A short esophagus ascends from the mouth towards Amb III where it joins the anterior stomach. At the apical part of the anterior stomach, a cluster of small, smooth, pointed pouches are located. As in *Cassidulus*, these pouches are directed towards the apex of Amb III and later divide to form two smaller clusters that are directed laterally, one towards IAmb 2, the other towards IAmb 3. The pouches are wedged between the upper coil of the digestive tract and are not in close proximity to the apical part of the test in Amb III. The number of pouches in an adult specimen of *Echinolampas depressa *was found to be approximately eight to twelve on each side.

#### Neolampadidae

The digestive tract of the single specimen of *Neolampas rostellata *(Figure 6D) that could be analyzed in the course of this study is characterized by the presence of the anterior stomach in Amb III. The short esophagus ascends from the mouth towards the anterior stomach, joining it in Amb III. A considerable dilation or pouch can be found branching off the anterior stomach towards IAmb 2. On the apical side of this structure, another knob-like dilation or pouch can be found. Its surface consists of several smooth, finger-like folds that adhere to each other.

#### Clypeasteridae

The anterior stomach of all species within the genus *Clypeaster *that have been analyzed so far is located in Amb III and IAmb 3. The short esophagus, originating from the top of Aristotle's lantern, is directed towards Amb III where it joins the anterior stomach. Distal to this junction, a grape-like cluster of pouches is present both in juvenile specimens of *Clypeaster europacificus *and adult specimens of *Clypeaster annandalei, Clypeaster destinatus *(Figure 6E) (Mortensen 71 regards the former two species as synonymous), *Clypeaster humilis*, *Clypeaster rarispinus *(Figure 6F), *Clypeaster reticulatus *(Figure 9O), and *Clypeaster rosaceus*. This cluster extends along the anterior stomach towards the anterior part of the animal in Amb III, and consists of dozens of smooth, grape-like nodules. Koehler [51: 27] described this structure as a "glande intestinale".

#### Arachnoididae

The anterior stomach in *Arachnoides placenta *(Figure 6G, H) is located in Amb III and reaches into Amb 2. A short esophagus connects the pharynx with the anterior stomach in Amb III. Between the central oral-aboral axis and the anterior tip of the test in Amb III, a cluster of pouches shaped like bunches of grapes extends laterally from the anterior stomach into IAmb 2. This cluster is present both in juvenile (Figure 6G) and adult specimens (Figure 6H), although its relative size seems to increase with age. The surface of each of the numerous individual pouches is smooth; some are more elongated than others. In adult specimens (ca. 10 cm test diameter), the entire cluster may attain a length of approximately 2 cm. Koehler [51: 27] described this structure as a "glande intestinale".

#### Laganidae

The anterior stomach in *Laganum bonani, Laganum decagonale*, *Laganum depressum*, *Laganum joubini*, *Laganum laganum *(Figures 7A, 9P), as well as in *Peronella lesueuri *and *Peronella orbicularis *(Figure 7B) is located in Amb III and IAmb 2, in some species extending well into Amb II. A short and broad esophagus reaches from the surface of Aristotle's lantern towards the anterior stomach in IAmb 2. The antero-lateral edge of the anterior stomach is slightly lobate in some species, particularly so in *Laganum laganum*. No particular dilation or pouch of the anterior stomach was observed in members of this taxon. Mortensen [71: 246] stated that in the Laganidae "...the intestinal gland appears to be lacking completely, in contradistinction to the Clypeastrids proper and *Arachnoides*."

#### Fibulariidae

The digestive tract of *Echinocyamus pusillus *(Figure 7C) is characterized by the presence of the anterior stomach at the border of Amb II and IAmb 2. The antero-lateral edge of the anterior stomach is smooth. The short esophagus connects the pharynx from the apical surface of Aristotle's lantern to the anterior stomach in IAmb 2. Dilations or pouches are absent from the anterior stomach.

#### Rotulidae

The anterior stomach of *Rotula deciesdigitata *(Figure 9Q) is located between Amb II and Amb III. A short esophagus spans from the surface of Aristotle's lantern towards the anterior stomach located in IAmb 2. The antero-lateral edge of the anterior stomach is smooth. Koehler [53] described the anterior stomach of *Rotula deciesdigitata *as considerably enlarged in comparison to the rest of the stomach. No dilations or pouches could be observed in the anterior stomach. Recent analysis of the subgenus *Fibulariella *(*sensu *Mortensen [71]) indicates that it is most closely related to the rotulids, not the fibulariids [72]. Accordingly, we report here that in *Fibulariella acuta *(Figure 7D), the configuration of the anterior stomach is similar to that in *Rotula.*

#### Echinarachniidae

The anterior stomach in *Echinarachnius parma *(Figures 7E, F; 9R) is located between Amb II and III. A short, broad esophagus connects the pharynx with the anterior stomach in IAmb 2. The abaxial edge of most parts of the stomach is characterized by the presence of a conspicuously frilled zone (Figure 7F) that can reach from IAmb 2 as far as IAmb 4. No particular dilation or pouch of the anterior stomach was observed.

#### Dendrasteridae

Data on the internal anatomy of dendrasterid sand dollars are available for *Scaphechinus griseus, Scaphechinus mirabilis, Scaphechinus tenuis *(Figure 7G), *Dendraster excentricus *(Figure 7H), and *Sinaechinocyamus mai *(Figure 7I). The anterior stomach is located between IAmb 2 and Amb III. A short, broad esophagus connects the pharynx with the anterior stomach at the border of Amb III and IAmb 2. Dilations or pouches of the anterior stomach could not be found. According to Reisman [55: 8], the stomach of *Dendraster excentricus *"is differentiated into a narrow, brown, fluted outer region and a wide mustard-yellow less fluted inner region."

#### Astriclypeidae

The digestive tract in *Astriclypeus manni *and *Echinodiscus auritus *(Figure 7J) is characterized by the presence of an anterior stomach located in IAmb 2 and Amb III. The short esophagus connects the pharynx with the anterior stomach in Amb III. The abaxial edge of most parts of the stomach consists of a smooth to frilled area that reaches from Amb III as far as Amb V. No particular dilation or pouch was observed in members of this taxon.

#### Mellitidae

The anterior stomach in *Encope stokesii *(Figure 7K), *Leodia sexiesperforata*, and *Mellita quinquiesperforata *(Figure 7L) is located in IAmb 2 and Amb III. A short esophagus connects pharynx with anterior stomach at the border of IAmb 2 and Amb III. The abaxial edge of parts of the stomach is characterized by the presence of a conspicuously frilled zone (Figure 7L) that may reach from IAmb 2 as far as Amb IV. The anterior stomach is devoid of any dilations or pouches.

### Atelostomata

Members of the following families all belong to a monophyletic taxon, the Atelostomata. All are characterized by the absence of Aristotle's lantern during all ontogenetic stages. Furthermore, the esophagus is directed towards the posterior part of the animal, i.e. IAmb 5, where it curves counter-clockwise until about IAmb 1, the branching-off point of the large primary siphon. Whether the subsequent part of the digestive tract from IAmb 1 to Amb III is derived from the esophagus or the stomach is currently a matter of debate. In the present study, we assume that the point at which the primary siphon branches off marks the end of the esophagus, implying that the anterior stomach has stretched from Amb III towards IAmb 1. This in turn creates a digestive tract area not present in non-atelostomate taxa. Histological and ultrastructural data support this view, but such data are patchy and currently available only for a limited number of taxa [4,8,9,12,69].

#### Corystidae

The anterior stomach in *Corystus relictus *(Figure 8A) extends from IAmb 1 until about IAmb 3. A large pouch is located on top part of the anterior stomach and extends clockwise from IAmb 3 until IAmb 5. Its connection with the anterior stomach is located in IAmb 3. The surface of the pouch is smooth.

#### Urechinidae

The anterior stomach in *Antrechinus nordenskjoldi *(Figure 8B) and *Urechinus naresianus *(Figure 8C, D) extends from IAmb 1 until about IAmb 3. A large pouch is located on top of the first part of the anterior stomach, extending from its connection to the former in Amb III clockwise towards IAmb 1. The surface of the pouch may be smooth (Figure 8B) or slightly lobate (Figure 8C). Mortensen [62: 42] states, that a "well developed diverticulum" is present in *Urechinus naresianus*.

#### Plexechinidae

The anterior stomach of *Plexechinus aoteanus *is characterized by the presence of a large pouch that connects to the underlying anterior stomach in Amb III.

#### Pourtalesiidae

The digestive tract of *Pourtalesia jeffreysi *(Figure 8E), *Pourtalesia hispida *(Figure 8F), and *Pourtalesia wandeli *(Figure 9S) is characterized by an anterior stomach that stretches between IAmb 1 and IAmb3. A large pouch is located on top of the first part of the anterior stomach, pointing from its connection to the former in Amb III straight towards IAmb 5. The apical surface of the pouch is smooth, whereas its sides as well as the adoral surface are lobate. According to Mortensen [62: 62], "the blind diverticulum is well developed, lobate" in the Pourtalesiidae.

#### Aeropsidae

The anterior stomach of *Aeropsis fulva *(Figure 8G) stretches approximately from IAmb 1 to IAmb 3. The esophagus is thin and joins the stomach presumably in IAmb 1. No particular dilation or pouch was observed in the vicinity of the anterior stomach. Although Agassiz [36] depicted a lateral view of the internal anatomy of *Aeropsis rostrata*, he unfortunately did not specifically mention the absence or presence of a pouch in the respective area of the digestive tract.

#### Hemiasteridae

The anterior stomach in *Hemiaster expergitus *(Figure 8H) is located between IAmb 1 and IAmb 3. A large pouch is located on top of the first part of the anterior stomach, pointing from its connection to the former in Amb III straight towards IAmb 5. The apical surface of the pouch is smooth, whereas its sides as well as the adoral surface are lobate. Koehler [63] mentions a long and only very slightly lobate pouch to be present at the same location in *Hemiaster hickmanni*.

#### Palaeopneustidae

In his account on various spatangoid species, Chesher [64] described the internal anatomy of *Spatangus purpureus *(see Spatangidae below), also mentioning the presence of a large pouch on top of the anterior stomach. For *Paleopneustes cristatus *and *Paleopneustes tholoformis *he noted that their internal anatomy largely resembled that of *Spatangus purpureus*, implying that there is a pouch present on top of the anterior stomach in these two palaeopneustid species as well.

#### Prenasteridae

Similar to his observations on palaeoneustid species, Chesher [64] noted that the internal anatomy of *Prenaster enodatus *largely resembled that of *Spatangus purpureus*, implying that there is a pouch present on top of the anterior stomach in this species as well.

#### Schizasteridae

The anterior stomach in *Schizaster canaliferus*, *Abatus cavernosus *(Figure 9T), *Abatus cordatus*, *Brisaster antarcticus*, *Brisaster fragilis*, and *Brisaster latifrons *(Figure 8I) is located between IAmb 1 and IAmb 3. A large pouch is located on top of the first part of the anterior stomach, pointing from its connection to the former in Amb III straight towards IAmb 5. The apical surface of the pouch is smooth, whereas its sides as well as the adoral surface are lobate. However, Koehler [9] described the pouch in *Schizaster canaliferus *as having simple, flat, and smooth walls. Later [63], he described the internal anatomy of other schizasterids and noted that the digestive tract in *Hypselaster kempi *is similar to the digestive tract in *Hemiaster hickmanni*, including the presence of a large pouch on top of the anterior stomach. On the other hand, Koehler [63] mentioned that there is no gastric caecum (= pouch in this description) in *Aceste ovata*.

#### Brissidae

The anterior stomach in *Brissus agassizii, Brissus unicolor *and *Meoma ventricosa *(Figure 8J, K) is located between IAmb 1 and IAmb 3. A large pouch is located on top of the first part of the anterior stomach, pointing from its connection to the former in IAmb 3 towards IAmb 5 in an oblique manner. The apical surface of the pouch is smooth, whereas its sides as well as the aboral surface are lobate. According to Chesher [67: 99], the pouch is a "highly vascularized, thin, convoluted sac which occupies a major portion of the coelom between intestine and the gonads. Sand does not enter the caecum." Agassiz [13: 677] gave a differing description of the anterior stomach in another brissid, *Metalia sternalis*: "...at the junction of the esophagus with the alimentary canal proper is found a cluster of small diverticula resembling those of *Rhynchopygus*, and not a single large diverticulum as in *Spatangus *proper." (*Rhynchopygus *= *Rhyncholampas *in the present article, see *Cassidulidae*).

#### Brissopsidae

The anterior stomach in *Brissopsis lyrifera *(Figure 8L) is located between IAmb 1 and IAmb 3. A large pouch is located on top of the first part of the anterior stomach, pointing from its connection to the former in IAmb 3 towards IAmb 5 in an oblique form. The apical surface of the pouch is smooth, whereas its sides as well as the adoral surface are lobate. According to Chesher [64], the following taxa closely resemble *Brissopsis lyrifera *with regard to internal anatomy, implying the presence of a pouch on top of the anterior stomach: *Brissopsis alta*, *Brissopsis atlantica*, *Brissopsis elongata*, and *Brissopsis mediterranea.*

#### Loveniidae

The anterior stomach in *Echinocardium cordatum *(Figure 8M) is located between IAmb 1 and IAmb 3. A large pouch is located on top of the first part of the anterior stomach, pointing from its connection to the former in IAmb 3 towards IAmb 5 in an oblique form. The apical surface of the pouch is smooth, whereas its sides as well as the adoral surface are lobate. According to De Ridder & Jangoux [12: 338], the anterior stomach of *Echinocardium cordatum *is characterized by the presence of "a large, translucent, turgid (fluid-filled), and non-contractile triangular pouch". Their analyses reveal that "no muscular sphincter is associated with the caecal opening, but a small prominence of dense connective tissue occurs at the level of the caecal slit. This prominence locally brings both faces of the caecal slit closer to each other, the opening never being tightly closed". Koehler [9] briefly described the internal anatomy of *Echinocardium cordatum *as well as *Echinocardium flavescens *and noted the presence of a large pouch with flat, simple, and smooth walls. Furthermore, Holland & Ghiselin [4] mentioned the presence of a large pouch atop the anterior stomach in *Lovenia subcarinata*.

#### Spatangidae

The anterior stomach in *Spatangus purpureus *(Figures 1A, C; 8 N, O) is located between IAmb 1 and IAmb 3. A large pouch is located on top of the first part of the anterior stomach, pointing from its connection to the former in IAmb 3 towards IAmb 5 in an oblique form. The apical surface of the pouch is smooth, whereas its sides as well as the adoral surface are lobate. Several authors described the pouch on top of the anterior stomach in *Spatangus purpureus *[8,9,13,14,17,18]. For example, Agassiz [13: 677] described the pouch as a "...huge diverticulum, trending upwards and towards the posterior extremity...", while Koehler [9] noted that the connection between the anterior stomach and the pouch appeared as a narrow elliptical orifice. He also mentioned the presence of numerous transverse folds in the walls of the pouch. Finally, Henry [17: 1318] stated that "...l'intestin du *Spatangus *est absolument bourré de sable et de coquilles fines, le caecum, au contraire, ne contient pas un grain de sable...". Data on the internal morphology of spatangids are available for two additional species, *Plethotaenia angularis *and *Plethotaenia spatangoides*: according to Chesher [64], their digestive tract anatomy closely resembles that of *Spatangus purpureus*, indicating the presence of a large pouch. MRI data reveal the presence of a smaller pouch atop the anterior stomach in *Pseudomaretia alta*. In this species, the pouch is connected to the exterior part of the anterior stomach in IAmb 3 and extends in an oblique form towards Amb II.

#### Maretiidae

Holland & Ghiselin [4] described the presence of a large pouch on top of the anterior stomach in *Maretia planulata*.

#### Asterostomatidae

The anterior stomach in *Heterobrissus niasicus *(Figure 8P) is located between IAmb 1 and IAmb 3. A large pouch is located atop the first part of the anterior stomach, pointing from its connection to the former in IAmb 3 towards IAmb 5 in an oblique form. The apical surface of the pouch is smooth, whereas its sides as well as the adoral surface are lobate. Wagner [69: 35] described the pouch in *Heterobrissus niasicus *in detail and noted that "...das (erste) Divertikel ist ein breiter Blindsack, der an der Außenseite der unteren Darmwindung im Radius III entspringt..." and "...es ist von unten nach oben abgeplattet, und seine Oberfläche ist von zahlreichen Falten bedeckt...". Koehler [63] briefly mentioned the internal organization of *Heterobrissus hemingi *and stated - based on Wagner's detailed description - that it is very similar to that in *Heterobrissus niasicus*. He furthermore noted the presence of a long, slim pouch on top of the anterior stomach in *Elipneustes denudatus*, whose general internal anatomy also closely resembled that of *Heterobrissus niasicus*.

## Discussion

Our conclusions are possible through comprehensive analyses of 168 sea urchin species representing almost all extant sea urchin families. This approach entails the combined use of invasive and non-invasive techniques, which permits to reconsider data provided in publications dating back almost 200 years. However, in many cases our observations had to be based upon single specimens due to the scarcity of certain species, especially those from the deep sea. It should be noted that the exact delineation of digestive tract structures such as the esophagus, stomach, intestine, and rectum is still under debate. Holland & Ghiselin [4] homologized substructures based on histological analyses. However, Jensen [6] mentioned - similar to Lewis' observations on *Diadema antillarum *[24] - that she had found valves within the digestive tract that would permit recognition of unequivocal homologies of sea urchin digestive tract compartments. Unfortunately, her data have never been published, so we base our designations primarily on Holland & Ghiselin's scheme [4].

### Criteria employed to homologize substructures of the sea urchin anterior stomach

In this section, we present a number of homology hypotheses that apply to all sea urchin taxa analyzed in this study: (i) presence or absence of festoons and of a gastric caecum in ambulacrum III; (ii) shape and size of the gastric caecum; (iii) mesenterial suspension of the anterior stomach and any substructures; and (iv) integration of the anterior stomach and any substructures into the haemal system of the digestive tract. The following sections include generalisations that are necessary in order to reveal the underlying homologies. However, we are aware that a certain degree of intra-species variability present in sea urchins could result in slightly differing conclusions. A condensed compilation of the findings presented in this section is available in Table 4.

#### (i) Absence or presence of festoons and of a gastric caecum in the anterior stomach

The entire stomach is always more or less festooned in "regular" sea urchins (Figure 9A-K), whereas in irregular sea urchins these vertical inflections are absent (Figure 9L-T). In "regular" and basal irregular sea urchin species, the anterior stomach is always located in Amb III or in Amb III and its adjoining interambulacra. In contrast, in the Atelostomata (Figure 9S, T), the anterior stomach extends from about Amb I to at least IAmb 3, although the exact homologies are still a matter of debate [4,6,12,15]. The number of festoons in Amb III may vary from none in the Irregularia, a single one in the Cidaroida (at the border of Amb III and IAmb3), to two in the "regular" Euechinoidea (at the borders of Amb III and IAmb 2 as well as Amb III and IAmb 3). This finding implies that the Cidaroida generally possess nine stomach festoons in total, whereas the "regular" Euechinoidea possess ten stomach festoons. Apart from the additional festoon in Amb III, the "regular" Euechinoidea deviate from the cidaroid scheme by the presence of a more or less developed dilation lateral to the additional festoon located at the border of Amb III and IAmb 2. It is this dilation that in the Irregularia is present in the form of a pouch, and we therefore homologize these structures, designating them from here on with the term "gastric caecum". This potential homology was also briefly mentioned by Koehler [9].

Of specific interest are those sea urchin taxa that lack a gastric caecum entirely or in which this structure can be found either in a reduced state or fused with adjoining structures. The single echinothurioid species so far known to lack the additional festoon as well as the gastric caecum is *Asthenosoma ijimai*, as depicted by Agassiz & Clark [32]. Since this description deviates from that of other species within the Echinothuriidae, it might be possible that the conspicuous lateral outcrop of the intestine in Amb III (Figure 3G) could actually be a part of the anterior stomach. Although the morphology of the esophagus in *Chaetodiadema pallidum *(Figure 4D) closely resembles that in other diadematid species, the stomach resembles that of cidaroids with respect to festoon number (nine) and gastric caecum (absent) in Amb III. Since this observation diverges from that in other diadematids, it needs to be verified in additional material. The digestive tract in arbacioids and salenioids is unlike those of most other "regular" sea urchin species because of its largely un-festooned, flat aspect that resembles the situation found in irregular sea urchins. Although it is difficult to differentiate individual festoons and the gastric caecum macroscopically in the Arbacioida and Salenioida, mesenterial strands connecting the digestive tract with the test clearly reveal the presence of these structures.

The apparent lack of a gastric caecum in the derived Clypeasteroida (Laganidae onwards, see Figure 2) is notable. Koehler [63] also mentioned the absence of such a structure in the derived clypeasteroids that he had termed the "glande intestinale" based on his analyses of the Clypeasteridae and Arachnoididae. However, he rejected a potential homology between this structure and the gastric caecum which he had previously observed in spatangoid taxa. Based on our data, we do not follow Koehler and instead consider the gastric caecum to be present in at least the two basal-most extant clypeasteroid taxa, the Clypeasteridae and the Arachnoididae. Whether the frilled zone (or sacculated area) at the abaxial edge of most parts of the stomach in certain derived clypeasteroid taxa must be considered a derivative of the gastric caecum needs to be assessed using comparative histological analyses on freshly fixed specimens. Our preliminary analyses using museum specimens were not successful due to the state of the material. Histological or even ultrastructural inferences might also be needed to determine whether the gastric caecum could have been internalized in taxa that lack both the gastric caecum as well as the sacculated area such as the Laganidae, the Fibulariidae, and the Rotulidae. Two further taxa within the Irregularia in which a gastric caecum appears to be absent are the two spatangoid genera *Aeropsis *and *Aceste*. No histological data are currently available for these taxa, and unfortunately not much is known regarding the biology of these deep-sea echinoids. So far, these two genera are the only known to lack the prominent gastric caecum that seems to be a characteristic feature of all other members of the Atelostomata.

#### (ii) Shape and size of the gastric caecum

The gastric caecum observed in the "regular" as well as the irregular Euechinoidea varies greatly in shape and size. In "regular" species, this structure is most prominent in taxa such as the Echinothurioida (e.g. Figure 3D) or the Diadematidae (e.g. Figure 4C). In the latter taxon, its orientation has additionally shifted from a lateral towards an adaxial position. However, a large gastric caecum can also be found in basal echinacean taxa such as *Glyptocidaris crenularis *(Figure 4E) and *Stomopneustes variolaris *(Figure 4F), its position lateral to the additional festoon in Amb III resembling the situation in the Echinothurioida rather than that in the Diadematidae. A small lateral dilation may be present in the Cidaroida as well, but based on the above mentioned homology hypothesis, we rule out any evolutionary relationship of this structure with the gastric caecum present in the Euechinoidea. Whether the cidaroid dilation has to be seen as the precursor (or the successor) of the additional festoon in Amb III encountered in "regular" euechinoids is impossible to determine based on the currently available data alone.

Although a gastric caecum is present in the more derived taxa of the Echinacea such as the Arbacioida, the Salenioida, and the Temnopleuroida, its exact size and shape are difficult to determine because it is largely fused with the adjoining additional festoon in Amb III. The distributional patterns of shape and size of the gastric caecum are more complex within the taxon Echinoida. Here, in the Parasaleniidae, Echinometridae, Strongylocentrotidae, and in part also the Toxopneustidae, the morphology of the anterior stomach closely resembles that found in *Stomopneustes variolaris *(Figures 4, 5, and 9). In contrast, the anterior stomach in the Parechinidae, Echinidae, and in part also in the Toxopneustidae is characterized by a gastric caecum that is largely fused with the additional festoon in Amb III, and which therefore more closely resembles the situation encountered in the Temnopleuroida.

**Figure 11:**
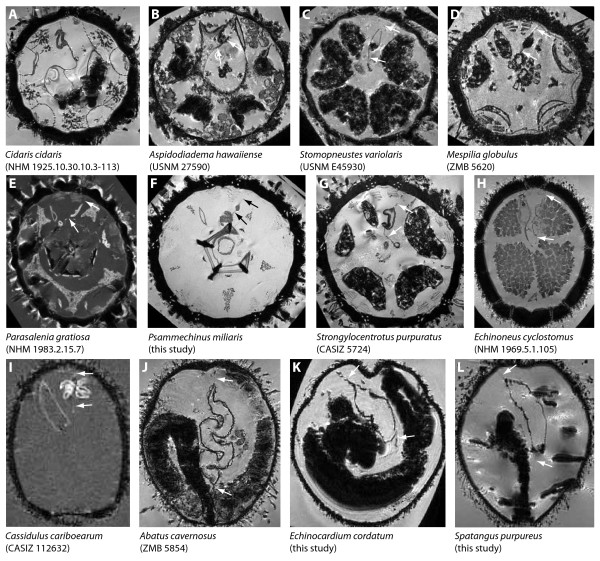
**Homology of the sea urchin gastric caecum based on its integration into the mesenterial system, in particularl the dorso-ventral mesentery, as a primary criterion**. Virtual horizontal sections based on MRI scans of Cidaridae (A), Aspidodiadematidae (B), Stomopneustidae (C), Temnopleuridae (D), Parasaleniidae (E), Parechinidae (F), Strongylocentrotidae (G), Echinoneidae (H), Cassidulidae (I), Schizasteridae (J), Loveniidae (K), and Spatangidae (L). The gastric caecum - where present - is attached to esophagus, axial complex, and the test through the dorso-ventral mesentery (arrows). In the Cidaroida (A), the dorso-ventral mesentery attaches to the single festoon present in ambulacrum III. In the more derived spatangoid and certain holasteroid taxa, this mesentery is shifted away from its original insertion near ambulacrum III towards interambulacrum 3, resulting in an oblique position of the gastric caecum (K, L). Note that the gastric caecum, in contrast to the rest of the digestive tract, is always free of sediment grains in the burrowing irregular taxa (H-L). Not to scale.

A rather common feature of the gastric caecum in the Irregularia is its position at the outer edge of the anterior stomach. However, despite this trait, the greatest variability in size and shape of the gastric caecum can be observed in irregular sea urchins. Here, the gastric caecum can either be absent (as in the derived Clypeasteroida and certain Spatangoida) or present in the form of multiple small nodules (basal Clypeasteroida), as multiple finger-like pouches (certain Cassiduloida), as well as in the form of a single smooth or lobate pouch that may extend from about a third (e.g. *Echinoneus*, Figure 9L) to more than half of the specimen's entire test length (e.g. *Abatus*, Figure 9T). In addition, the connections of the gastric caecum to the anterior stomach may be either through a broad canal or through a slit-like opening which may aid in preventing sediment grains from entering - the gastric caecum of the irregular sea urchins observed in this study always had a liquid content (Figure 11H-L). Furthermore, the macroscopic appearance of the gastric caecum in most Irregularia is always that of a thin-walled, semi-transparent pouch which in most cases easily permits distinction of this part of the stomach.

Interestingly, the description by Agassiz [13] of the gastric caecum in *Metalia sternalis*, a derived spatangoid, closely resembles that of the gastric caecum found in cassiduloids. Since we have never found a similarly shaped gastric caecum in any other spatangoid, we presume that Agassiz must have erred when determining the species. Although Koehler [9] noted differences in the extent to which the walls of the gastric caecum are covered in folds within spatangoids, we were not able to identify any meaningful pattern within this taxon. However, the gastric caecum in the Cassidulidae, Echinolampadidae, Neolampadidae, basal Clypeasteroida, as well as certain Holasteroida is smooth-walled; we consider this condition to be a derived feature, based on the presence of a lobate gastric caecum in the more basal irregular taxa Echinoneidae (Figure 9L) and Apatopygidae (Figure 6B).

Analyses of different developmental stages of specimens from five species, *Paracentrotus lividus*, *Psammechinus miliaris*, *Echinometra viridis*, *Echinoneus cyclostomus *and *Arachnoides placenta *reveal that the additional festoon as well as the gastric caecum are present already in juvenile specimens. The observed distinct architecture of the anterior stomach must therefore be considered as a development-independent feature.

#### (iii) Mesenterial suspension of the anterior stomach

Of particular interest for the elucidation of gastric caecum homologies are the various mesenteries that are involved in the suspension of the anterior stomach. The abaxial edge of the entire stomach is usually attached to the test through a more or less continuous mesenterial sheet that also follows the vertical inflections of the festoons, in turn permitting to identify individual festoons using primarily MRI. A second mesentery, termed the dorso-ventral mesentery, attaches the esophagus to the test as well as to the axial complex [31]. The gastric caecum is partly surrounded by and, therefore, included within this mesentery. Except for the Cidaroida (which do not possess the additional festoon in Amb III as well as the gastric caecum) and atelostomates (detailed below), the dorso-ventral mesentery extends from the central oral-aboral axis towards the test near the border of IAmb 2 and Amb III (Figure 11B-J). In the more derived Spatangoida (Brissidae onwards, see Figure 2), this connection has shifted counter-clockwise at least as far as IAmb 3, resulting in an oblique form of the entire gastric caecum (Figure 11K, L) as opposed to a straight form seen in most other irregular sea urchins (Figure 11H-J). A similar observation can be made in certain holasteroid species, especially in *Corystus *(Figure 8A) and *Urechinus *(Figure 8C).

Further suspension of the anterior stomach can be observed in the Diadematidae, where the "ends" of the lower gut loop (i.e. the stomach) are fused by a strong mesentery (Figure 4A). A similar - clearly convergent - development can be observed in the Clypeasteridae (Figure 6E).

#### (iv) Integration of the anterior stomach into the digestive tract haemal system

**Figure 12:**
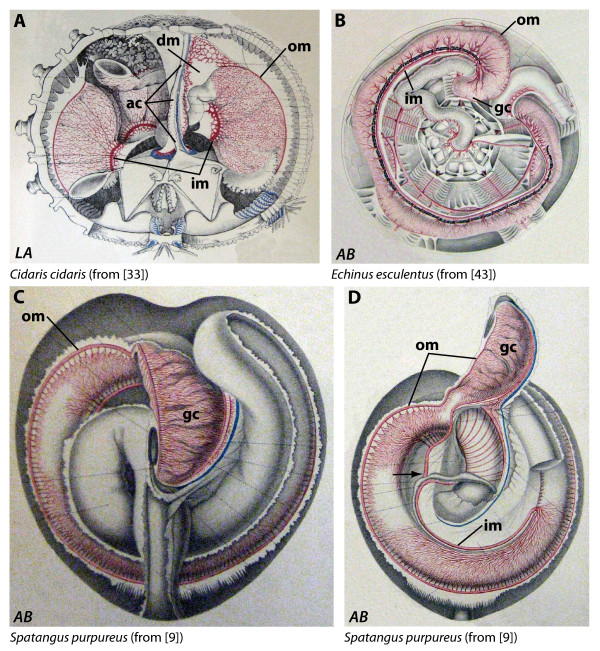
**Homology of the sea urchin gastric caecum based on its integration into the haemal system of the digestive tract as a primary criterion**. Schematic representations of the digestive tract haemal system in Cidaridae (A), Echinidae (B), and Spatangidae (C, D). The sea urchin stomach is accompanied by an inner (im) as well as an outer (om) marginal haemal duct. The outer marginal haemal duct sends out branches towards the dorso-ventral mesentery (dm) which is connected to the axial complex (ac) (A). The gastric caecum (gc) - if present (B-D) - is well-integrated into the haemal system. The black arrow in (D) depicts the conspicuous side branch of the inner marginal haemal duct present in presumably all spatangoids and potentially further irregular sea urchin taxa. *AB *= aboral view, *LA *= lateral view. Not to scale.

Sea urchins are characterized by a relatively complex haemal system [9,22,33,43]. The stomach is well integrated into this system and is supplied by an inner as well as outer marginal haemal duct. The inner marginal haemal duct ascends from the esophageal haemal ring along the esophagus and borders the adaxial edge of the anterior stomach (Figure 12A). The outer marginal haemal duct borders the abaxial edge of the anterior stomach and sends out branches towards the dorso-ventral mesentery which is connected to the axial complex and its haemal spaces. The outer marginal haemal duct is present in this form presumably in all "regular" sea urchins (Figure 12B). In the Spatangoida, the outer marginal haemal duct is located on the aboral side of the gastric caecum (Figure 12C). The inner marginal haemal duct, however, underwent considerable changes, although the precise timing of these events is difficult to trace based on the present data alone. These changes have led to the evolution of a side-branch of the inner marginal haemal duct which descends from the main branch in order to supply the adoral side of the gastric caecum. Such a novel side-branch can be found in the Clypeasteroida (Figure 6E, F) as well as the Spatangoida (Figure 12D). We were not able to identify this structure in the Echinoneoida, the Cassiduloida, or the Holasteroida and are therefore unsure about its origins. As indicated above, a number of authors elaborated on the complex anatomy of the sea urchin haemal system and so far the Cidaroida [33], the Echinothurioida [34], the Arbacioida [22], the Echinoida [22,23,43,48], the Temnopleuroida [41], the Clypeasteroida [40,55], and the Spatangoida [8,9,12,64,66,69] have been covered. Since the present study did not reveal any novel data on this subject, we will not consider the haemal system of the digestive tract any further.

### Evolutionary origin of the highly specialized gastric caecum

As detailed in the previous section, we believe the gastric caecum found in most irregular sea urchins to be homologous with the dilation of the anterior stomach observed in the "regular" Euechinoidea. This dilation can therefore be seen as the precursor of the spatangoid gastric caecum, proposing a solution for the question of the evolutionary origin of this enigmatic organ.

If we consider the presence of the additional festoon as well as the gastric caecum in Amb III as a synapomorphy of the Euechinoidea, in turn the question of the origin of these structures arises. Since the extant sister group to the Euechinoidea, the Cidaroida, possesses only a single festoon in Amb III, we are not able to determine precisely what the condition in the ancestor of both taxa might have been. To clarify this aspect, analyses of mesenterial strand imprints upon the internal part of the test, potentially visible in extant and well-preserved fossil specimens (*sensu *Roman [73]), might be helpful. It seems obvious to assume that the ancestor of both Cidaroida and Euechinoidea should have possessed two festoons in Amb III, given the otherwise strong pentameric arrangement of the internal anatomy of "regular" sea urchins. This notion is supported in part by the comparatively large anterior stomach in two basal cidaroid taxa, the Histocidaridae and the Ctenocidaridae, which could hint at a stepwise reduction of the additional festoon within the Cidaroida. But then the question arises as to why the Cidaroida had lost this part of the anterior stomach, since there is no structure occupying the void between the first (in Amb III) and the last (in Amb II) festoon of the stomach that can be found in all cidaroids - Stewart's organs as well as large gonads are present in the basal Euechinoidea as well. Further studies on the general homology of mesenteries in sea urchins might help to answer this question.

A probable explanation for the presence of additional digestive tract elements in the basal Euechinoidea such as the Echinothurioida or Diadematidae might be that their feeding habits differ from those of the Cidaroida. Most "regular" sea urchins are considered omnivorous, although cidaroids display a certain degree of carnivory, whereas most other "regular" echinoids are considered omnivorous with herbivorous habits [5]. In addition, the "regular" euechinoids are capable of forming so-called food pellets, primarily using their buccal cavity and pharynx. To what degree the additional festoon as well as the gastric caecum might play a role in processing mucous-enclosed food pellets needs to be addressed in future studies. One might hypothesize that water circulation throughout the digestive tract is facilitated when the gut content is packed into pellets which in turn could contribute to the feeding process and its dynamics. Extended comparative histological analyses will be necessary to elucidate any potential usefulness of the gastric caecum in "regular" euechinoids with regard to feeding or nutrient absorption.

Once present, the gastric caecum was modified during evolution, with a number of significant changes in "regular" and irregular sea urchins becoming apparent: (i) reduction of stomach festoons and fusion of the additional festoon with the lateral dilation (Arbacioida, Salenioida); (ii) fusion of the additional festoon with the lateral dilation (Temnopleuroida, some Echinoida); (iii) specialization of the lateral dilation into a thin-walled, fluid-filled, semi-transparent pouch always free of sediment (Irregularia); (iv) division of the single pouch into numerous finger-like sacs and further reduction into grape-like nodules (some Cassiduloida and Clypeasteroida); (v) enlargement of the pouch combined with a shift of the opening towards the anterior (some Cassiduloida, Atelostomata), and finally (vi) shifting of the axis of orientation of the pouch from an axis along Amb III-IAmb 5 to IAmb 3-Amb I (some Holasteroida and Spatangoida).

The drastic changes observed within the Irregularia, including the wholesale reduction of the gastric caecum in some taxa, presumably correlate with feeding habits and lifestyle of the various taxa. Although the precise function of the gastric caecum remains unknown, it seems obvious that a role in the digestive process can be assumed. Irregular sea urchins are primarily infaunal deposit feeders and are known to inhabit various types of sediment [74]. Future studies will therefore have to address the question to what extent the shape and size of gastric caeca might correlate with sediment type and feeding habits.

### Phylogenetic implications

From our anatomical descriptions it becomes clear that the absence or presence as well as shape and relative size of the gastric caecum characterizes individual sea urchin taxa. The compilation of our findings presented here suggests a number of phylogenetic conclusions. This includes support for the monophyly of the Cidaroida, the Euechinoidea, as well as the Irregularia. The divergent shapes of the gastric caecum in cassiduloid families suggest a potential non-monophyly of the Cassiduloida, a conclusion reached by others (e.g. [75,76]). The loss of a gastric caecum could furthermore support the monophyly of the currently unnamed taxon comprising the Laganina and the Scutellina, with additional support for the Scutellina being obtained through the presence of a conspicuously frilled zone at the abaxial edge of most parts of the stomach. Loss of the gastric caecum in the spatangoid genera *Aeropsis *and *Aceste *could be indicative of the need to group these taxa more closely together. The presence of a flattened stomach without festoons is in support of a potentially close relationship between the Salenioida and the Irregularia. The morphology of the anterior stomach in sea urchins provides a number of characters with phylogenetic signal (see Table 4 for a character matrix):

1. Anterior stomach extends into ambulacra I and II: absent (0) (Figure 3A); present (1) (Figure 8G).

2. Stomach festoons: absent (0) (Figure 9L); present (1) (Figure 9C).

3. Number of festoons in ambulacrum III: one (0) (Figure 3C); two (1) (Figure 3D).

4. Festoons in ambulacrum III: separate (0) (Figure 4F); fused (1) (Figure 4G).

5. Stomach sacculated at abaxial edge: absent (0) (Figure 7C); present (1) (Figure 7L).

6. Gastric caecum: absent (0) (Figure 3B); present (1) (Figure 3E).

7. Shape of gastric caecum: dilation (0) (Figure 5H); single pouch (1) (Figure 6A); multiple pouches (2) (Figure 6C).

8. Shape of multiple pouches: finger-like (0) (Figure 6C); grape-shaped (1) (Figure 6E).

9. Mesenterial suspension of gastric caecum: along axis from ambulacrum III to interambulacrum 5 (0) (Figure 11J); along axis from interambulacrum 3 to ambulacrum I (1) (Figure 11K).

10. Position of connection(s) between gastric caecum and anterior stomach with regard to the gastric caecum: median (0) (Figure 6A); anteriorly (1) (Figure 8I).

11. Macroscopic appearance of gastric caecum: undifferentiated from stomach (0) (Figure 5B); thin-walled, semi-transparent (1) (Figure 8M).

12. Stomach ends fused in ambulacrum III: absent (0) (Figure 4E); present (1) (Figure 4A).

13. Position of dilation in ambulacrum III: lateral (0) (Figure 4E); adaxial (1) (Figure 4A)

**Table 4:** Compilation of the primary morphological findings of this study related to the sea urchin anterior stomach in the form of a character matrix.

Taxon/Character	1	2	3	4	5	6	7	8	9	10	11	12	13
Histocidaridae	0	1	0	-	0	0	-	-	-	-	-	0	-

Ctenocidaridae	0	1	0	-	0	0	-	-	-	-	-	0	-

Cidaridae	0	1	0	-	0	0	-	-	-	-	-	0	-

Psychocidaridae	0	1	0	-	0	0	-	-	-	-	-	0	-

Phormosomatidae	0	1	1	0	0	1	0	-	0	-	0	0	0

Echinothuriidae	0	1	1	0	0	1	0	-	0	-	0	0	0

Pedinidae	0	1	1	0/1	0	1	0	-	0	-	0	0	0

Micropygidae	0	1	1	0	0	1	0	-	0	-	0	0	0

Aspidodiadematidae	0	1	1	0	0	1	0	-	0	-	0	0	0

Diadematidae	0	1	1	0	0	1	0	-	0	-	0	1	1

Glyptocidaridae	0	1	1	0	0	1	0	-	0	-	0	0	0

Stomopneustidae	0	1	1	0	0	1	0	-	0	-	0	0	0

Arbaciidae	0	1	1	1	0	1	0	-	0	-	0	0	0

Saleniidae	0	1	1	1	0	1	0	-	0	-	0	0	0

Temnopleuridae	0	1	1	1	0	1	0	-	0	-	0	0	0

Trigonocidaridae	0	1	1	1	0	1	0	-	0	-	0	0	0

Parasaleniidae	0	1	1	0	0	1	0	-	0	-	0	0	0

Parechinidae	0	1	1	1	0	1	0	-	0	-	0	0	0

Echinidae	0	1	1	1	0	1	0	-	0	-	0	0	0

Echinometridae	0	1	1	0	0	1	0	-	0	-	0	0	0

Strongylocentrotidae	0	1	1	0/1	0	1	0	-	0	-	0	0	0

Toxopneustidae	0	1	1	0/1	0	1	0	-	0	-	0	0	0

Echinoneidae	0	0	-	-	0	1	1	-	0	0	1	0	-

Apatopygidae	0	0	-	-	0	1	1	-	0	1	1	0	-

Cassidulidae	0	0	-	-	0	1	2	0	0	?	1	0	-

Echinolampadidae	0	0	-	-	0	1	2	0	0	?	1	0	-

Neolampadidae	0	0	-	-	0	1	2	0	0	?	1	0	-

Clypeasteridae	0	0	-	-	0	1	2	1	0	?	1	1	-

Arachnoididae	0	0	-	-	0	1	2	1	0	?	1	0	-

Laganidae	0	0	-	-	0	0	-	-	-	-	-	0	-

Fibulariidae	0	0	-	-	0	0	-	-	-	-	-	0	-

Rotulidae	0	0	-	-	0	0	-	-	-	-	-	0	-

Echinarachniidae	0	0	-	-	1	0	-	-	-	-	-	0	-

Dendrasteridae	0	0	-	-	1	0	-	-	-	-	-	0	-

Astriclypeidae	0	0	-	-	1	0	-	-	-	-	-	0	-

Mellitidae	0	0	-	-	1	0	-	-	-	-	-	0	-

Corystidae	1	0	-	-	0	1	1	-	1	1	1	0	-

Urechinidae	1	0	-	-	0	1	1	-	1	1	1	0	-

Plexechinidae	1	0	-	-	0	1	1	-	1	1	1	0	-

Pourtalesiidae	1	0	-	-	0	1	1	-	0/1	1	1	0	-

Aeropsidae	1	0	-	-	0	0	-	-	-	-	-	0	-

Hemiasteridae	1	0	-	-	0	1	1	-	0	1	1	0	-

Paleopneustidae	1	0	-	-	0	1	1	-	?	?	?	0	-

Prenasteridae	1	0	-	-	0	1	1	-	?	?	?	0	-

Schizasteridae	1	0	-	-	0	0/1	-/1	-	-/0	-/1	-/1	0	-

Brissidae	1	0	-	-	0	1	1	-	1	1	1	0	-

Brissopsidae	1	0	-	-	0	1	1	-	1	1	1	0	-

Loveniidae	1	0	-	-	0	1	1	-	1	1	1	0	-

Spatangidae	1	0	-	-	0	1	1	-	1	1	1	0	-

Maretiidae	1	0	-	-	0	1	1	-	1	1	1	0	-

Asterostomatidae	1	0	-	-	0	1	1	-	1	1	1	0	-

The results have been condensed in order to provide a general overview of the phylogenetically informative characters (13 in total) that could be derived from our analysis. See the "Phylogenetic implications" section of the "Discussion" for an explanation of the characters used. - = not applicable, ? = data not available.

## Conclusions

The present study constitutes a contribution towards better understanding of digestive tract structures in sea urchins from an evolutionary perspective. Our data permit identification of the relevant transformational stages in the course of the evolution of the highly specialized gastric caecum present in the derived Spatangoida. According to our findings, the sea urchin gastric caecum constitutes a synapomorphy of the Euechinoidea. Its occurrence in "regular" euechinoids is linked to the presence of an additional festoon of the anterior stomach in Amb III. Both structures, the additional festoon and the gastric caecum, are absent in the Cidaroida. Since the degree of specialization of the gastric caecum is most pronounced in the predominantly sediment-burrowing irregular taxa, we hypothesize that its evolution is closely linked to the development of more elaborate infaunal lifestyles. We provide a comprehensive study of the origin and evolutionary plasticity of a conspicuous digestive tract structure, the gastric caecum, in a major taxon of the extant invertebrate macrozoobenthos.

## Methods

### Species used in this study

The specimens referred to in this study are listed in Tables 2 and 3 together with information on the systematic classification of each species, the source of the data used in the study, specimen ID where applicable, and literature references. The freshly fixed specimens were collected in Elba (Italy), Heligoland (Germany), Sydney Harbour (Australia), Discovery Bay (Jamaica), New Caledonia (France) or were purchased from aquarium supply companies. The systematic classifications are based upon results obtained by [75-84].

### Dissection and photography

Dissection was performed on freshly fixed as well as museum specimens under direct observation through a stereo-microscope equipped with a digital camera for documentation.

### Magnetic resonance imaging

Magnetic resonance imaging was performed using the methods described in [25,85,86]. Imaging was carried out in Berlin and Würzburg, Germany using high-field small animal MRI scanners equipped with 7 T, 9.4 T, and 17.6 T super-conducting electromagnets, respectively. The resolution of the datasets acquired varied between ~(20 μm)^3 ^and (96 μm)^3 ^for 3D protocols and was 50 × 50 × 200 μm^3 ^or 78 × 78 × 300 μm^3 ^for the 2D protocols employed. Tables 2 and 3 list the resolutions achieved for every species analyzed by MRI. Image processing was carried out using ImageJ 1.42q (NIH, USA) and its Volume Viewer plugin. Unfortunately, we are currently unable to provide the raw image data online due to the lack of a centralized repository for digital morphological data (see [87] for discussion).

### 3D modelling and visualization

3D image reconstruction and modelling were performed using the methods described in [25,85,86]. The interactive 3D models in Figure 10 were embedded using the Adobe 3D Reviewer software (part of the Adobe Acrobat 9 Pro Extended suite) according to procedures described in [88-90]. All figures were arranged and assembled using Adobe Photoshop CS3 and Adobe Illustrator CS3.

## List of abbreviations used

2D: two-dimensional; 3D: three-dimensional; AAD: Australian Antarctic Division, Kingston, Australia; AM: Australian Museum, Sydney, Australia; CASIZ: California Academy of Sciences Invertebrate Zoology, San Francisco, USA; MNHN: Muséum Nationale de la Histoire Naturelle, Paris, France; MRI: magnetic resonance imaging; NHM: Natural History Museum, London, United Kingdom; NHMW: Naturhistorisches Museum, Wien, Austria; NIWA: National Institute of Water & Atmospheric Research, Wellington, New Zealand; USNM: National Museum of Natural History, Washington D.C., USA; ZMB: Systematische Zoologie am Museum für Naturkunde, Berlin, Germany; ZMH: Zoologisches Institut und Museum, Hamburg, Germany; ZSM: Zoologische Staatssammlung, München, Germany.

## Competing interests

The authors declare that they have no competing interests.

## Authors' contributions

AZ designed and coordinated the study, carried out dissections and specimen photography, performed literature search, prepared specimens for MRI scanning, scanned specimens, carried out 3D modelling, and wrote the manuscript. RM carried out dissections and specimen photography. GR performed dissection as well as literature search. CD carried out dissection, literature search and histological analyses. CD and RM contributed to writing the manuscript. All authors read and approved of the final version.
